# Increased expression of cathepsin C in airway epithelia exacerbates airway remodeling in asthma

**DOI:** 10.1172/jci.insight.181219

**Published:** 2024-11-22

**Authors:** Lin Yuan, Qingwu Qin, Ye Yao, Long Chen, Huijun Liu, Xizi Du, Ming Ji, Xinyu Wu, Weijie Wang, Qiuyan Qin, Yang Xiang, Bei Qing, Xiangping Qu, Ming Yang, Xiaoqun Qin, Zhenkun Xia, Chi Liu

**Affiliations:** 1 Department of Physiology, School of Basic Medicine Science,; 2 Basic and Clinical Research Laboratory of Major Respiratory Diseases, and; 3 Department of Pulmonary and Critical Care Medicine, Second Xiangya Hospital, Central South University, Changsha, China.; 4 Functional Center, School of Basic Medical Sciences, Xinjiang Medical University, Urumqi, Xinjiang, China.; 5 Department of Thoracic Surgery, Second Xiangya Hospital, Central South University, Changsha, Hunan, China.; 6 Centre for Asthma and Respiratory Disease, School of Biomedical Sciences and Pharmacy, Faculty of Health and Medicine, University of Newcastle and Hunter Medical Research Institute, Callaghan, New South Wales, Australia.

**Keywords:** Pulmonology, Therapeutics, Asthma, Respiration

## Abstract

Airway remodeling is a critical factor determining the pathogenesis and treatment sensitivity of severe asthma (SA) or uncontrolled asthma (UA). The activation of epithelial-mesenchymal trophic units (EMTUs) regulated by airway epithelial cells (AECs) has been proven to induce airway remodeling directly. However, the triggers for EMTU activation and the underlying mechanism of airway remodeling are not fully elucidated. Here, we screened the differentially expressed gene cathepsin C (CTSC; also known as dipeptidyl peptidase 1 [DPP-1]) in epithelia of patients with SA and UA using RNA-sequencing data and further verified the increased expression of CTSC in induced sputum of patients with asthma, which was positively correlated with severity and airway remodeling. Moreover, direct instillation of exogenous CTSC induced airway remodeling. Genetic inhibition of CTSC suppressed EMTU activation and airway remodeling in two asthma models with airway remodeling. Mechanistically, increased secretion of CTSC from AECs induced EMTU activation through the p38-mediated pathway, further inducing airway remodeling. Meanwhile, inhibition of CTSC also reduced the infiltration of inflammatory cells and the production of inflammatory factors in the lungs of asthmatic mice. Consequently, targeting CTSC with compound AZD7986 protected against airway inflammation, EMTU activation, and remodeling in the asthma model. Based on the dual effects of CTSC on airway inflammation and remodeling, CTSC is a potential biomarker and therapeutic target for SA or UA.

## Abstract 2

Increased secretion of CTSC in airway epithelia induces airway remodeling in asthma through enhanced activation of EMTU by the p38-mediated signaling pathway. Based on the dual role of CTSC in airway inflammation and airway remodeling, the study provides a new treatment for severe asthma or uncontrolled asthma.

## Graphical abstract

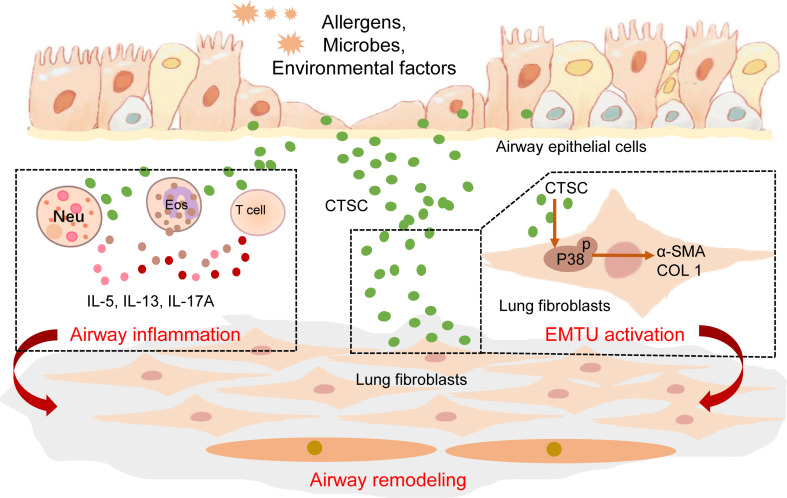

## Introduction

Asthma is a heterogeneous clinical syndrome characterized by chronic airway inflammation, airway remodeling, and airway hyperresponsiveness (1). Although inhaled corticosteroids or combined use of them with long-acting β-receptor agonists have greatly improved the clinical symptoms of patients with asthma, long-term use of antiinflammatory drugs has limited effects on improving lung function and preventing asthma exacerbations in patients (2, 3). Of note, airway remodeling is an important determinant of asthma severity and treatment sensitivity as well as a critical clinicopathologic characteristic of severe asthma (SA) or uncontrolled asthma (UA) (4, 5). Specifically, with the increase of asthma severity, the degree of airway remodeling often deteriorates (6). From childhood to adulthood, airway remodeling can directly induce irreversible respiratory obstruction and airway hyperresponsiveness, which is independent of airway inflammation and persists even after the subsides of airway inflammation (7–12). Moreover, targeted antiinflammatory therapy alone cannot prevent or alleviate the occurrence and development of airway remodeling (13). Therefore, airway remodeling is a critical factor affecting the clinicopathological process and therapeutic effect of patients with asthma. However, the underlying mechanisms of airway remodeling in asthma remain obscure.

Traditional viewpoints hold that airway remodeling and inflammation are interdependent (14). However, the latest studies have proven that airway remodeling appears in the early stage of asthma and is not influenced by steroid therapy (15). Our previous studies have also confirmed that airway remodeling can occur independently or in parallel with airway inflammation, indicating the noninflammatory mechanisms for airway remodeling in patients with asthma (10, 16, 17). Consistent with this, increasing evidence confirms that airway epithelial cells (AECs) are important inducers of the initiation and development of airway remodeling (14, 18). After exposure to noxious stimuli, damaged AECs can directly activate fibroblasts by releasing various inflammatory and growth factors (e.g., TGF-β1 and EGF), leading to structural damage of airway and ultimately inducing airway remodeling (18, 19). This epithelial regulation of mesenchymal tissue is defined as the activation of epithelial-mesenchymal trophic units (EMTUs) (20, 21). The activation of EMTUs is proven to be the central event of airway remodeling after epithelial injury, which is independent of airway inflammation (21, 22). However, the underlying mechanism of EMTU activation and airway remodeling after epithelial injury in asthma is far from clear.

In this study, the differentially expressed genes (DEGs) in the airway epithelia of patients with asthma were identified from Gene Expression Omnibus (GEO) databases (https://www.ncbi.nlm.nih.gov/gds). Using bioinformatics analysis screening, we found that the expression of cathepsin C (CTSC; also known as dipeptidyl peptidase 1 [DPP-1]) increased markedly, which is most pertinent to airway remodeling of patients with asthma. On this basis, the expression of CTSC in the induced sputum of patients with asthma was verified, and the correlation between the expression of CTSC and lung function/airway remodeling parameters was analyzed, separately. Moreover, a recombinant mouse CSTC protein–challenged (rmCTSC-challenged) model and two different asthma models with airway remodeling were employed to investigate the effect of CTSC on airway remodeling, EMTU activation, and airway inflammation. Finally, the underpinning molecular mechanism of airway remodeling induced by epithelial CTSC was explored in vitro and the corresponding intervention strategy was implemented in vivo.

## Results

### The expression of CTSC increases markedly in the airway epithelia of patients with SA or UA.

Gene expression profiles from the GEO database (accession GSE19187 and GSE63142) were used to identify DEGs between samples from healthy individuals acting as controls (HCs) and SA or UA samples. 1,769 DEGs from GSE19187 and 12,45 DEGs from GSE63142 were screened out (Supplemental Figure 1, A and B; supplemental material available online with this article; https://doi.org/10.1172/jci.insight.181219DS1), and there were 300 coincident DEGs (Supplemental Figure 1C). Pathway and process enrichment analysis was carried out to analyze the function and pathway of the 300 DEGs (Supplemental Figure 1D and Supplemental Table 1). To further capture the relationship among different terms, a subset of enriched terms was selected and rendered as a network plot (Supplemental Figure 1, E and F). Two remodeling-related pathways, NABA MATRISOME ASSOCIATED and NABA ECM REGULATORS, ranked second and third respectively (Supplemental Table 1). In particular, CTSC ranked first in both of these two pathways. Moreover, the increased expression of CTSC was also detected in patients with SA or UA from the two datasets (Supplemental Figure 1, G and H). These data suggest the involvement of the increased CTSC in AECs in the pathogenesis of airway remodeling in patients with asthma.

### The expression level of CTSC is positively related to the degree of airway remodeling in patients with asthma.

**Figure 1:**
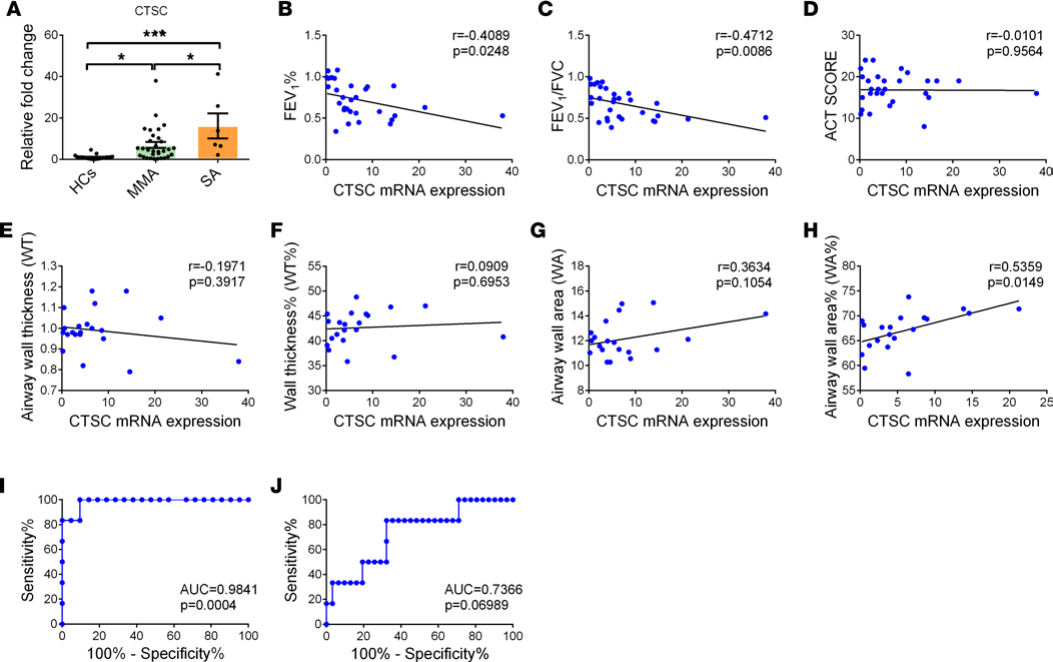
The expression of CTSC is related to asthma severity in patients. (**A**) The mRNA expression of CTSC in the induced sputum of patients with asthma and HCs. Data are presented as mean ± SEM. One-way ANOVA followed by Tukey’s post hoc test was used. (**B** and **C**) Pearson’s correlation analysis between CTSC expression and pulmonary function in patients with asthma. FEV_1_, forced expiratory volume in 1 second; FVC, forced vital capacity. (**D**) Pearson’s correlation analysis between CTSC expression and ACT score in patients with asthma. (**E**–**H**) Pearson’s correlation analysis between CTSC expression and related parameters of airway remodeling in patients with asthma. (**I**) Receiver operating characteristic analysis for the expression of CTSC to discriminate HCs from patients with SA. (**J**) Receiver operating characteristic analysis for the expression of CTSC to discriminate patients with MMA from patients with SA. *P < 0.05; ***P < 0.001.

To further investigate the relationship between the level of CTSC and airway remodeling in asthma, the expression of CTSC was detected in the sputum samples from HCs, individuals with mild-to-moderate asthma (MMA), and individuals with SA from the Second Xiangya Hospital (Supplemental Table 2). Compared with HCs, the expression of CTSC increased markedly in patients with MMA and further increased in patients with SA (Figure 1A). Then, we analyzed the correlation between the level of CTSC and the related indicators of airway remodeling (including lung function, asthma control test [ACT] score, and airway CT parameters) in patients with asthma (Figure 1, B–H). Notably, the level of CTSC was negatively correlated with FEV_1_% and FEV_1_/FVC (Figure 1, B and C) and positively associated with the wall area and the percentage of wall area of patients with asthma (Figure 1, G and H). These results corroborate the notion that the increased expression of CTSC is closely related to the pathogenesis of airway remodeling in patients with asthma.

Based on the previous results, logistic regression was used to build a prediction model to evaluate whether the expression level of CTSC could distinguish HCs from patients with SA. Consistent with our predictions, the AUC value for the level of CTSC was 0.98 (Figure 1I). Moreover, the level of CTSC could also be used to predict MMA versus SA with AUC values of 0.74 (Figure 1J). These results indicate that the increased expression of CTSC is a potential biomarker of SA.

### Increased expression of CTSC induces airway remodeling in asthma models.

**Figure 2:**
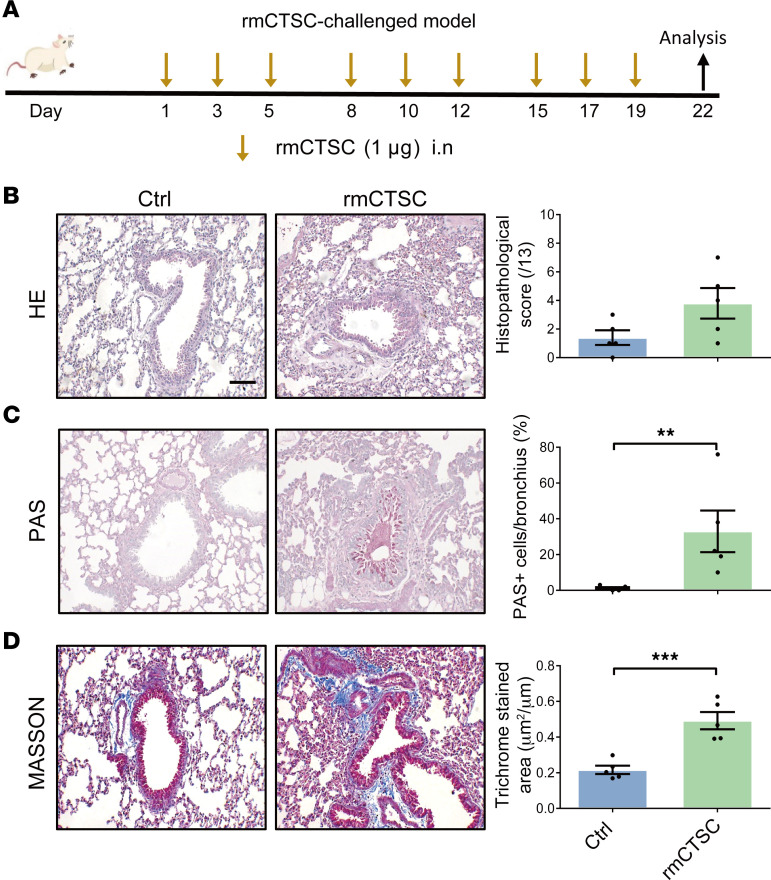
Exogenous CTSC promotes airway remodeling. (**A**) Schematic of the rmCTSC-challenged mouse model. (**B**) Representative lung sections and semiquantitative analysis of airway inflammation (*n* = 5; scale bar: 50 μm). Unpaired *t* test was used. (**C**) Representative lung sections and semiquantitative analysis of mucus production (*n* = 5; scale bar: 50 μm). Mann-Whitney *U* test was used. (**D**) Representative lung sections and semiquantitative analysis of peribronchial fibrosis (*n* = 5; scale bar: 50 μm). Unpaired *t* test was used. All data are presented as mean ± SEM. **P < 0.01; ***P < 0.001.

To investigate the specific effect of CTSC in airway remodeling, rmCTSC was used to characterize the effects of pulmonary CTSC in vivo (Figure 2A). Our results demonstrated that there was no marked change in airway inflammation after chronic administration of rmCTSC in WT mice (Figure 2B and Supplemental Figure 2, A–E). However, marked increases in mucus production and collagen deposition were observed in rmCTSC-challenged WT mice (Figure 2, C and D). These data provide evidence that the airway remodeling induced by CTSC is at least partially independent of airway inflammation.

**Figure 3:**
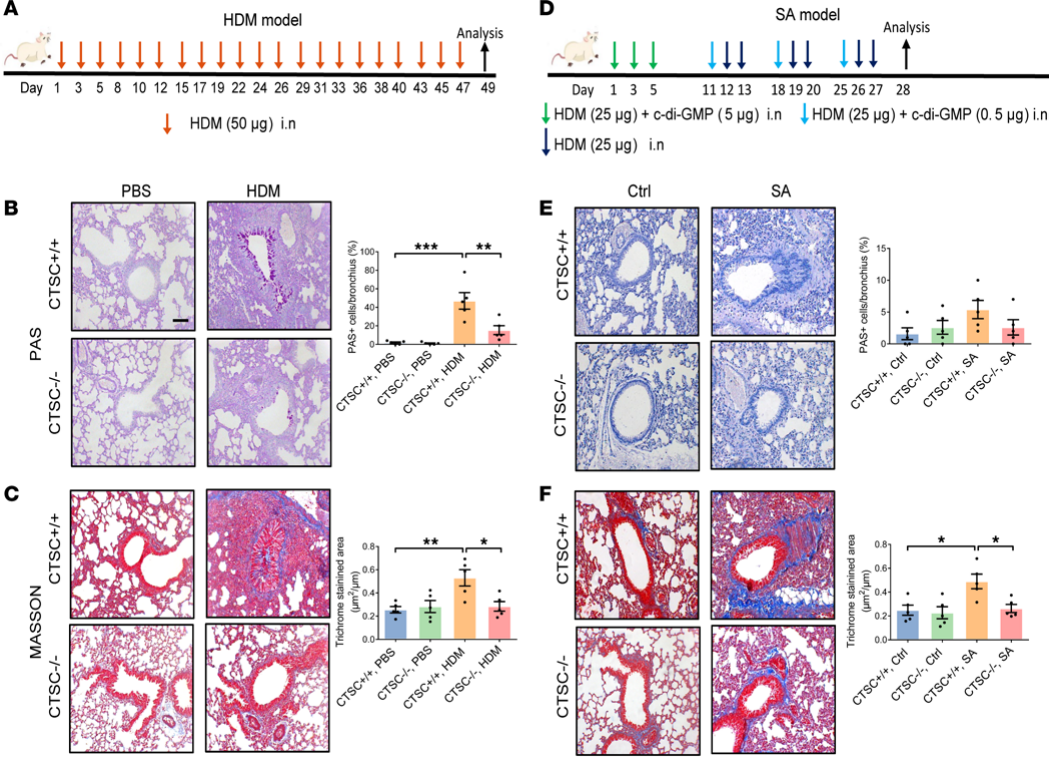
Airway remodeling is markedly alleviated in the absence of CTSC. (**A** and **D**) Schematic of the (**A**) HDM model and (**D**) SA model. (**B** and **E**) Representative lung sections and semiquantitative analysis of mucus production (*n* = 5; scale bar: 50 μm). (**C** and **F**) Representative lung sections and semiquantitative analysis of peribronchial fibrosis (*n* = 5; scale bar: 50 μm). All data are presented as mean ± SEM. *P < 0.05; **P < 0.01; ***P < 0.001 by 1-way ANOVA followed by Tukey’s post hoc test.

As direct instillation of CTSC resulted in airway remodeling, CTSC^–/–^ mice were also used to verify the role of CTSC in the pathogenesis of airway remodeling. Seven-week house dust mite–induced (HDM-induced) asthma model with airway remodeling was constructed (Figure 3A). Compared with control mice, 7-week HDM-stressed mice showed marked airway inflammation and airway remodeling that was characterized by thickening of basement membrane, increased hyperplasia of goblet cells, and exaggerated deposition of collagen in the small airway (Supplemental Figure 3, A–C). However, mucus production (Figure 3B and Supplemental Figure 4A) and collagen deposition (Figure 3C) were alleviated in CTSC^–/–^ mice compared with those in CTSC^+/+^ mice after HDM stress.

Furthermore, a SA model was also constructed to further observe the effect of CTSC on airway remodeling in SA (Figure 3D and Supplemental Figure 5, A–C). Although SA models in both CTSC^+/+^ and CTSC^–/–^ mice did not show an increase in mucus production (Figure 3E and Supplemental Figure 4B), alleviated collagen deposition was detected in the SA model of CTSC^–/–^ mice compared with that in CTSC^+/+^ mice (Figure 3F). These data demonstrate that the increased expression of CTSC aggravates airway remodeling in asthma models.

### Increased expression of CTSC in AECs promotes EMTU activation in asthma models with airway remodeling.

Previous results of gene expression profiles (GSE19187 and GSE63142) showed that the expression of CTSC increased markedly in the epithelial cells of patients with asthma (Supplemental Figure 1, G and H). In addition, the expression of CTSC in the lung and AECs was further evaluated by qPCR and immunohistochemistry, separately. Compared with the corresponding control group, increased expression of CTSC in the lung and AECs was detected in both the HDM-challenged group and SA group, respectively (Supplemental Figure 6, A–D). These results further indicated that the increased expression of CTSC in AECs is closely associated with airway remodeling in asthma.

**Figure 4:**
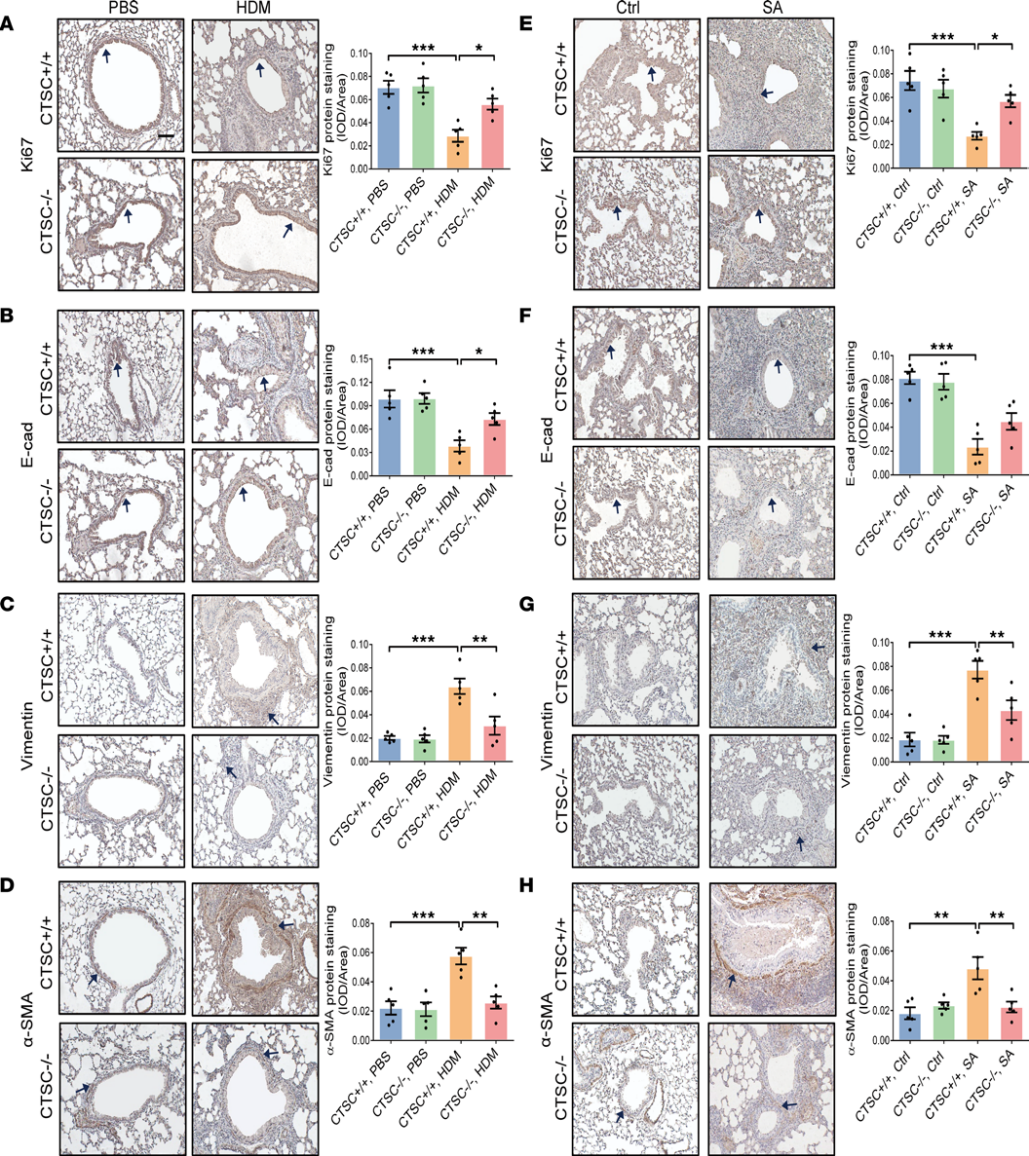
The activation of EMTU is markedly decreased in the absence of CTSC. (**A**–**D**) Representative immunohistochemistry images of lung tissue and semiquantitative analysis for (**A**) Ki67, (**B**) E-cad, (**C**) vimentin, and (**D**) α-SMA expression in a HDM-induced model (*n* = 5; scale bar: 50 μm). (**E**–**H**) Representative immunohistochemistry images of lung tissue and semiquantitative analysis for (**E**) Ki67, (**F**) E-cad, (**G**) vimentin, and (**H**) α-SMA expression in SA model (*n* = 5; scale bar: 50 μm). All data are presented as mean ± SEM. *P < 0.05; **P < 0.01; ***P < 0.001 by 1-way ANOVA followed by Tukey’s post hoc test.

Since CTSC-induced airway remodeling is at least partially independent of airway inflammation in vivo (Figure 2), and the activation of EMTU by AECs is considered a central event of airway remodeling (21), we speculated that the increased expression of CTSC in AECs may promote airway remodeling directly through increased activation of EMTU. Compared with CTSC^+/+^ mice, higher levels of Ki67 and E-cad in AECs and lower levels of vimentin and α-SMA around the bronchia were detected in CTSC^–/–^ mice after HDM exposure (Figure 4, A–D), indicating that EMTU activation was lessened. Similar results were also detected in SA model (Figure 4, E–H). These data provide evidence that increased CTSC in AECs enhances EMTU activation in patients with asthma.

### Increased expression of CTSC disrupts the wound repair ability of HBECs.

**Figure 5:**
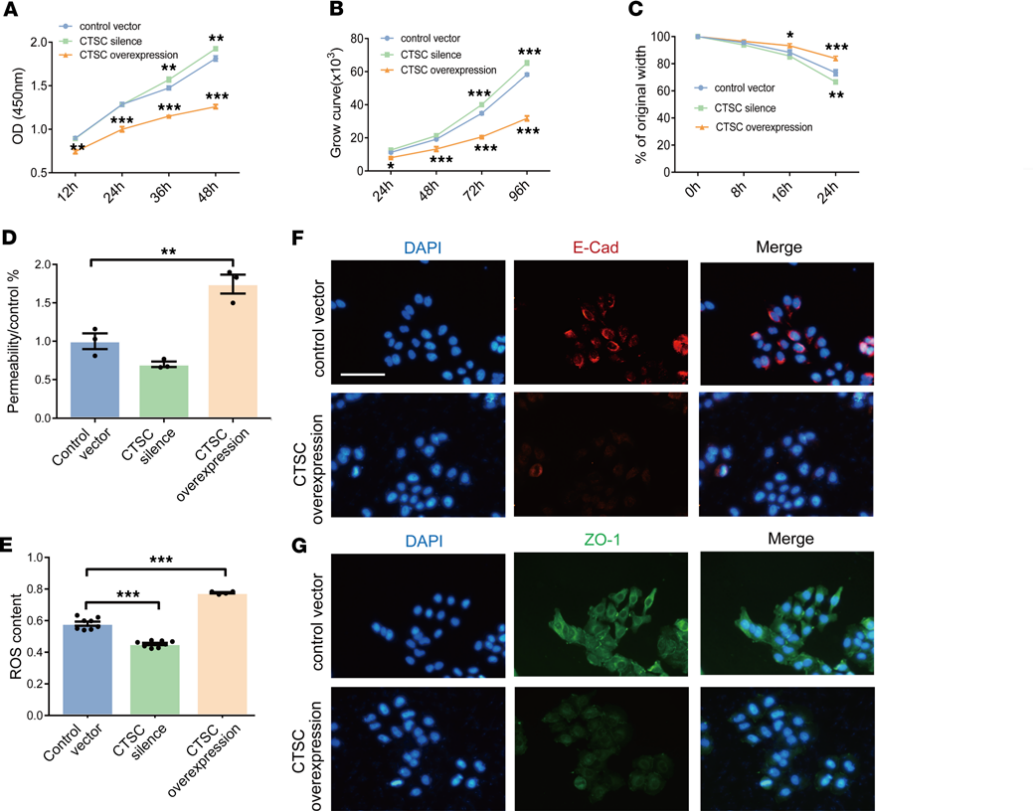
The increased expression of CTSC impairs the epithelial barrier function. (**A**) Cell proliferation analysis of HBECs after CSTC silencing and overexpression using CCK-8 assay (*n* = 5). Two-way ANOVA followed by Tukey’s post hoc test was used. (**B**) Proliferation curves of HBECs after CSTC silencing and overexpression (*n* = 4). Two-way ANOVA followed by Tukey’s post hoc test was used. (**C**) A scratch test for evaluating damage repair capability in HBECs (*n* = 3). Two-way ANOVA followed by Tukey’s post hoc test was used. (**D**) Effects of CTSC expression on the permeability of HBECs monolayers (*n* = 3). One-way ANOVA followed by Tukey’s post hoc test was used. (**E**) Effects of CTSC expression on ROS generation (*n* = 4–8). One-way ANOVA followed by Bonferroni’s post hoc test was used. (**F** and **G**) Representative immunofluorescence of HBECs stained for E-cad and ZO-1 after CSTC overexpression (*n* = 3; scale bar: 50 μm). All data are presented as mean ± SEM. *P < 0.05, **P < 0.01, ***P < 0.001.

Both clinical and animal studies demonstrated the increased expression of CTSC in AECs of patients with asthma. To further investigate the direct effect of CTSC on human bronchial epithelial cells (HBECs) in vitro, CTSC-silenced and overexpressed HBECs were constructed, respectively (Supplemental Figure 7, A and B). The results of CCK-8 and growth curve analyses demonstrated that the proliferation ability of HBECs was inhibited markedly in CTSC overexpressed group (Figure 5, A and B). Consistent with this, 24 hours after the initial scratch, a wider scratch was detected in CTSC-overexpressed HBECs, while slightly narrower scratch was detected in CTSC-silenced HBECs (Figure 5C). Moreover, the permeability coefficient of HBECs monolayers was increased and the antioxidative activity of HBECs was decreased in the CTSC-overexpressed cell group (Figure 5, D and E). Further analysis also demonstrated lower levels of cell-cell adhesion proteins (E-cad and ZO-1) in the CTSC-overexpressed cell group (Figure 5, F and G). Similar results were also verified in 16HBE14o- cells (Supplemental Figure 8, A–G). Combined, these data support the notion that increased expression of CTSC disrupts the wound repair ability of AECs.

### Increased secretion of CTSC in HBECs enhances EMTU activation through p38-mediated pathway.

**Figure 6:**
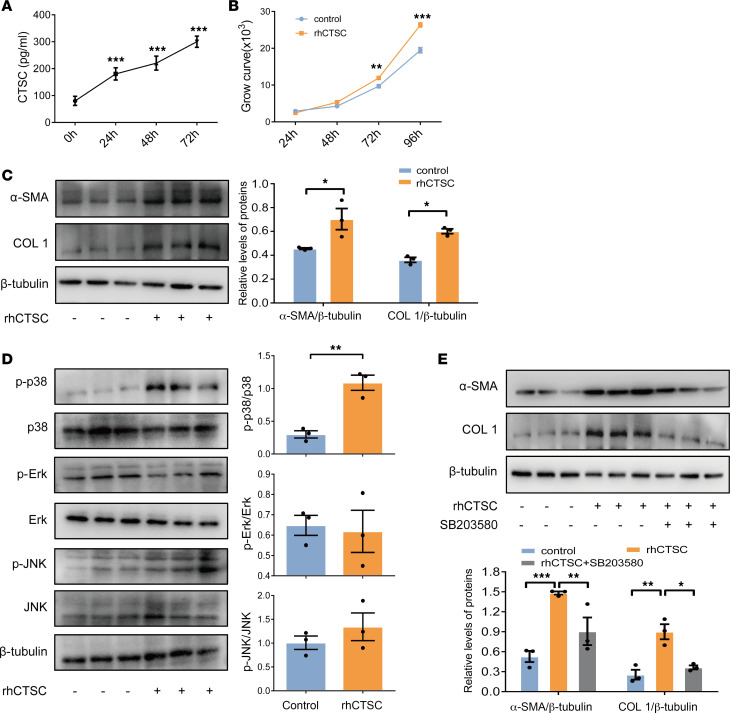
rhCTSC increases the activation of EMTU through activation of p38 pathway. (**A**) The secretion of CTSC in HBECs was detected by ELISA (*n* = 4). One-way ANOVA followed by Tukey’s post hoc test was used. (**B**) Proliferation curves of HLF-1 after rhCTSC stimulation (*n* = 4). Two-way ANOVA followed by Tukey’s post hoc test was used. (**C**) Expression of α-SMA and COL 1 protein was detected by Western blot (*n* = 3). Two-way ANOVA followed by Tukey’s post hoc test was used. (**D**) Expression of p38, p-p38, Erk, p-Erk, JNK, and p-JNK protein was detected by Western blot (*n* = 3). Unpaired *t* test was used. (**E**) HLF-1 were pretreated with p38 inhibitor SB203580 and then stimulated with rhCTSC. Expression of α-SMA and COL 1 protein was detected by Western blot (*n* = 3). Two-way ANOVA followed by Tukey’s post hoc test was used. All data are presented as mean ± SEM. *P < 0.05, **P < 0.01, ***P < 0.001.

CTSC protein contains a signal peptide and could be secreted by various cells (23). However, whether CTSC can be secreted from HBECs and the function of secretory CTSC are largely unclear. Of note, the content of CTSC in the supernatant of HBECs increased markedly after HDM stress, which was positively correlated with the duration of HDM stimulation (Figure 6A). These results indicated that HDM stressed–AECs may enhance EMTU activation by increased secretion of CTSC. To further elucidate the effect of HBECs-derived CTSC on EMTU activation in vitro, the proliferation and activation of HLF-1 was detected after stimulation with recombinant CTSC (rhCTSC). As expected, rhCTSC had a marked promoting effect on the proliferation and activation of HLF-1 (Figure 6, B and C). Taken together, these results indicate that the increased CTSC not only inhibits the wound repair ability of AECs, but also promotes EMTU activation through secretion.

The MAPK signaling pathway is an essential mechanism of fibroblast activation (24) that has been shown to be regulated by CTSC (25). Based on this, we speculated that the increased secretion of CTSC from HBECs may regulate the activation of fibroblasts through the MAPK pathway. Consistent with predictions, rhCTSC stimulation markedly enhanced the activation of the p38 pathway rather than the Erk pathway or JNK pathway in HLF-1 (Figure 6D). The p38 inhibitor (SB203580) could substantially reduce the activation of HLF-1 and collagen synthesis induced by rhCTSC (Figure 6E). These results suggest that the increased secretion of CTSC from AECs promotes EMTU activation partly through the p38-mediated pathway.

### Increased expression of CTSC induces airway inflammation in asthma models.

**Figure 7:**
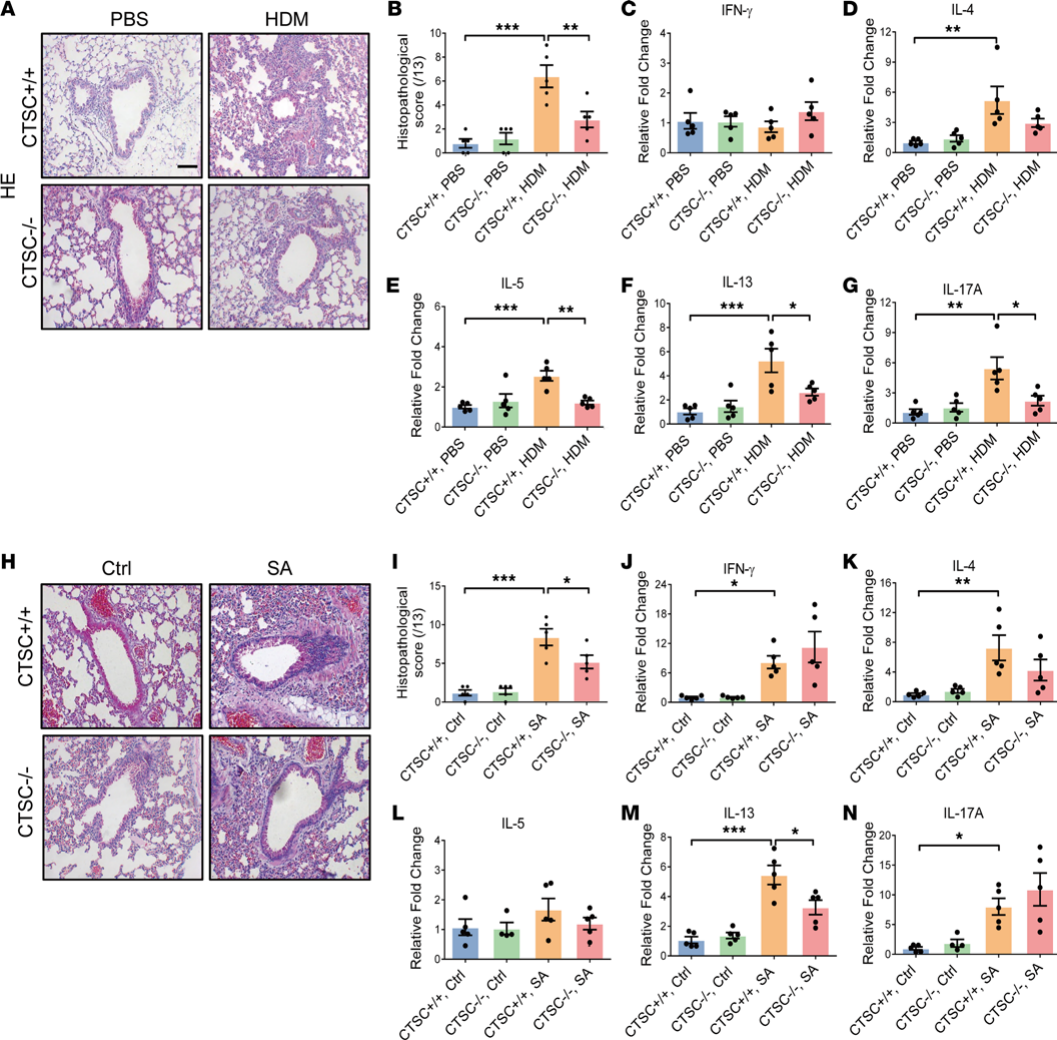
Airway inflammation is markedly alleviated in the absence of CTSC. (**A**) Representative lung sections and semiquantitative analysis of airway inflammation in a HDM model (*n* = 5; scale bar: 50 μm). (**B**–**G**) The levels of IFN-γ, IL-4, IL-5, IL-13, and IL-17A transcripts in lung tissue were examined by quantitative PCR in the HDM model (*n* = 5). (**H**) Representative lung sections and semiquantitative analysis of airway inflammation in a SA model (*n* = 5; scale bar: 50 μm). (**I**–**N**) The levels of IFN-γ, IL-4, IL-5, IL-13, and IL-17A transcripts in lung tissue were examined by quantitative PCR in the SA model (*n* = 4–5). All data are presented as mean ± SEM. *P < 0.05; **P < 0.01; ***P < 0.001 by 1-way ANOVA followed by Tukey’s post hoc test.

As airway remodeling and airway inflammation are bidirectional interactions in asthma (14), airway inflammation was also examined by H&E and qPCR in asthma models. Decreased inflammatory cells and inflammatory cytokines were detected in HDM-stressed CTSC^–/–^ mice (IL-5, IL-13, and IL-17A) compared with CTSC^+/+^ mice (Figure 7, A–G). Consistently, SA model of CTSC^–/–^ mice developed decreased inflammatory cells and inflammatory cytokines (IL-13) in lung tissues (Figure 7, H–N). These data indicate that the increased expression of CTSC induces airway inflammation, which may further aggravate airway remodeling in asthma models.

### Targeting CTSC through AZD7986 prevents airway remodeling and airway inflammation in the HDM-induced asthma model.

**Figure 8:**
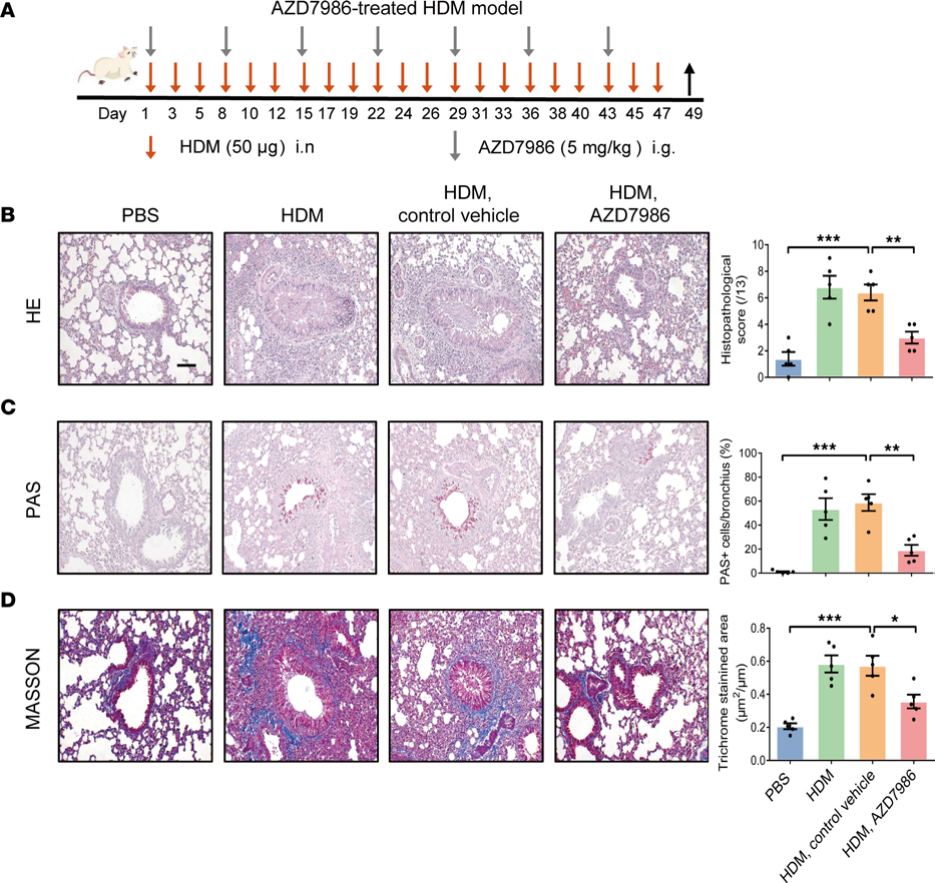
The administration of AZD7986 alleviates airway remodeling in a HDM-induced asthma model. (**A**) Schematic of the HDM model mice treated with AZD7986. (**B**) Representative lung sections and semiquantitative analysis of airway inflammation (*n* = 5; scale bar: 50 μm). (**C**) Representative lung sections and semiquantitative analysis of mucus production (*n* = 5; scale bar: 50 μm). (**D**) Representative lung sections and semiquantitative analysis of peribronchial fibrosis (*n* = 5; scale bar: 50 μm). All data are presented as mean ± SEM. *P < 0.05; **P < 0.01; ***P < 0.001 by 1-way ANOVA followed by Tukey’s post hoc test.

We further investigated the therapeutic potential of CTSC inhibitor (AZD7986) in the HDM-stressed model (Figure 8A). Similar to CTSC^–/–^ mice, marked reductions in lung inflammation (Figure 8B and Supplemental Figure 9, A–E), mucus production (Figure 8C), collagen deposition (Figure 8D), and EMTU activation (Supplemental Figure 10, A–D) were observed in mice pretreated with AZD7986 compared with vehicle control. These data confirmed the dual role of CTSC in airway remodeling and airway inflammation, which suggested that CTSC might serve as a potential treatment target for patients with asthma.

## Discussion

Asthma is a common chronic respiratory disease that is characterized by airway inflammation, airway hyperresponsiveness, reversible airway obstruction, and irreversible airway remodeling (26). It is particularly noteworthy that airway remodeling not only involves structural changes in the airway wall, but also leads to sustained or progressive damage to lung function of individuals with SA or UA (4, 27). However, there is a lack of effective treatment for airway remodeling in patients with asthma at present (13). In this study, our clinical results demonstrated that the expression of airway remodeling–related protein CTSC increased markedly in the AECs of patients with asthma, which was a potential biomarker of SA. The increased expression of CTSC induced airway remodeling through enhanced activation of EMTU that was mediated by the p38-signaling pathway. More than that, increased CTSC also contributed to airway inflammation, as genetic inhibition of CTSC reduced the infiltration of inflammatory cells and the production of inflammatory factors in the lungs of asthmatic mice. Consequently, targeting CTSC with compound AZD7986 effectively suppressed airway inflammation and remodeling simultaneously in the HDM-induced asthma model. These findings elucidate the molecular mechanisms of CTSC-induced airway remodeling and highlight CTSC as the potential therapeutic target for both airway inflammation and airway remodeling in patients with asthma.

AECs are the first cellular barrier to resist environmentally hazardous stimuli. Damaged AECs have been proven to drive the initiation and development of airway remodeling directly (14, 18). Therefore, exploring the pathogenesis of remodeling based on AECs may provide new directions for its treatment (22). In this study, using a bioinformatics approach, our results found that the increased expression of CTSC in epithelia was most pertinent to airway remodeling in patients with asthma. Moreover, compared with MMA or controlled asthma, the increase of CTSC in the airway or nasal epithelium of SA or UA was more marked. CTSC is one of the 8 cysteine cathepsin family members, localized in endolysosomal compartments (28). It is a lysosomal cysteine protease that is necessary for activating granule cell–associated serine proteases (29, 30). In particular, CTSC can also participate in the degradation of extracellular matrix (ECM), activation of growth factors, tissue remodeling, and cell growth or damage (31, 32). Our clinical results further demonstrated that the expression of CTSC was positively correlated with the parameters of airway remodeling in patients with asthma, which may be a potential predictive biomarker of patients with SA.

It has been proven that airway remodeling in patients with asthma has noninflammatory induction mechanisms (14). Our studies demonstrated that the increased expression of CTSC in the airway epithelia of patients with asthma could induce airway remodeling by activating EMTU in the absence of obvious inflammation, which is consistent with our previous research (33) and parallel studies (17, 34). For example, in preschool children with established SA, there is an increase in the mass of airway smooth muscle and thickening of the reticular basement, which is independent of inflammation (17, 34). Here, our study further provides evidence that airway remodeling can occur independently of airway inflammation, which may be the reason for the limited clinical effects of antiinflammatory glucocorticoid therapy in some patients with asthma.

It is noteworthy that CTSC protein contains a signal peptide that allows for conventional secretion (23, 35). However, little is known about the role of secretory CTSC from AECs in EMTU activation and airway remodeling. As MAPK pathways play an important role in proliferation and differentiation in fibroblasts (36, 37) and targeting MAPK pathways has been proven to be a treatment for pulmonary and renal fibrosis (38, 39), we investigated the effect of secretory CTSC on the barrier function of AECs and differentiation of lung fibroblasts and MAPK pathways. The relevant results showed that the increased expression of CTSC not only disrupted the barrier of AECs, but also was released into the lung microenvironment. Then, AEC-derived CTSC promoted EMTU activation through the p38-mediated pathway, ultimately inducing airway remodeling. These results show that CTSC is a critical mediator of EMTU activation and airway remodeling in multiple models of asthma.

It should also be noted that airway remodeling and airway inflammation are bidirectional interactions in asthma (14). Our results have shown that the increased expression of CTSC also promoted the infiltration of inflammatory cells and the production of inflammatory factors (e.g., IL-5, IL-13, and IL-17A) in the HDM-induced asthma model and SA model. Recurrent episodes or persistence of airway inflammation can further exacerbate airway remodeling (14), Thus, CTSC may also further participate in the regulation of airway remodeling through direction regulation of inflammatory factors or indirect effects on other inflammatory cells or structural cells in the lung (29, 40–42). Consistent with this, our unpublished research has also revealed that AEC-derived CTSC could stimulate the synthesis and release of CTSC from neutrophils, which suggests that, besides its direct promotion of EMTU activation and airway remodeling, AEC-derived CTSC can also exacerbate airway remodeling by modulating CTSC secretion in other inflammatory cells. In addition, our results have shown that neither CTSC gene knockout nor CTSC inhibitor could completely inhibit airway remodeling in asthma, indicating that other molecules, such as other family members of cysteine proteases (43), may also participate in the process of airway remodeling, which still requires a series of future studies. Meanwhile, our study found that direct instillation of a certain concentration of exogenous CTSC induced airway remodeling without inflammation. This may be due to the difference in activity or quantity of CTSC receptors, which leads to lung fibroblasts being more sensitive to secretory CTSC than inflammatory cells.

Based on the critical role of airway inflammation in the classification and treatment of asthma (44), finding targets with dual antiinflammatory and antiremodeling effects may be the future direction of asthma management. The dual effect of AZD7986 on airway inflammation and airway remodeling in asthma model suggests its promising application prospect in patients with asthma, especially those with airway remodeling. As a small-molecule, competitive, and reversible inhibitor of CTSC, AZD7986 (also called brensocatib) is being investigated in a phase III clinical trial (ASPEN; NCT04594369) in noncystic fibrosis bronchiectasis (NCFBE). Results from a phase II study in patients with NCFBE demonstrated that treatment with 10 or 25 mg brensocatib for 24 weeks was associated with improved clinical outcomes, including prolongation in the time to first exacerbation and reduction in the frequency of exacerbations (45). Although dental and skin adverse events were observed at doses of 10 mg and 25 mg brensocatib, these were not considered to prevent further clinical development (45, 46). Absolutely, more attention and investigation should be paid in future studies.

However, several limitations of the study should be acknowledged. First, the expression and secretion of CTSC in AECs or air-liquid interface from patients with asthma cannot be verified in the clinic owing to the difficulty of obtaining bronchial mucosal tissue in patients with asthma. Second, the dosage and administration mode of corticosteroids in patients with asthma may affect the expression of CTSC and its correlation with parameters of pulmonary function and CT data. Third, further research should be conducted on the effect of secretory CTSC from the other cell sources (e.g., macrophages, neutrophils, and mast cells) and other family members of cysteine protease in aggravating airway remodeling. In addition, the specific receptor for secretory CTSC in fibroblasts is still obscure.

In summary, this study has demonstrated that the increased expression of CTSC in the airway epithelia of patients with asthma induces airway remodeling through enhanced activation of EMTUs and aggravation of airway inflammation. Based on the dual effects of CTSC on airway inflammation and airway remodeling, CTSC may be a potential biomarker and therapeutic target for SA or UA.

## Methods

### Sex as a biological variable.

Both male and female participants were enrolled in this clinical cohort. Our study examined male and female mice, and the effect of sex differences on the findings is unknown.

### Microarray data.

The National Center for Biotechnology Information’s Gene Expression Omnibus (GEO) datasets were searched, using “asthma”, “epithelium,” and “epithelial cell” as keywords. The inclusion criteria included the following: (a) mRNA chip types; (b) grouping of HCs, asthma control, or severity; and (c) study published between 2011 and 2016. The exclusion criteria included the following: (a) lack of grouping of asthma control or severity and (b) gene expression data not being available. Then, the GSE19187 and GSE63142 gene expression profiles were screened and downloaded from the GEO database (47, 48). The GSE19187 dataset included 11 nasal epithelium samples of HCs, 7 nasal epithelial samples of controlled asthma, and 6 nasal epithelial samples of UA. In addition, the GSE63142 dataset included 27 bronchial epithelium samples of HCs, 72 bronchial epithelial samples of MMA, and 56 bronchial epithelial samples of SA.

The raw data were processed by Affy package in R language (49). Significance of differential expression was tested using R package-limma (50) and adjusted for multiple testing with Benjamini-Hochberg (51). Only the genes with adjusted *P* values of less than 0.05 and fold change of more than 1.2 were selected as DEGs. The volcano plot was also produced with R language.

### Pathway and process enrichment analysis.

To identify DEGs, Metascape online software (https://metascape.org/gp/index.html#/main/step1) was used to carry out pathway and process enrichment analysis with the following ontology sources: Kyoto Encyclopedia of Genes and Genomes (https://www.genome.jp/kegg/), Gene Ontology (https://geneontology.org/) biological processes, Reactome (https://reactome.org/) gene sets, Canonical Pathways (http://www.broadinstitute.org/gsea/msigdb/collection_details.jsp#CP), and CORUM (https://mips.helmholtz-muenchen.de/corum/). All genes in the genome were used as the enrichment background. Terms with a *P* value of less than 0.01, minimum count of 3, and enrichment factor of more than 1.5 (enrichment factor is the ratio between observed count and the count expected by chance) were collected and grouped into clusters based on their membership similarities. Kappa scores were used as the similarity metric in hierarchical clustering on the enriched terms and then subtrees with similarity greater than 0.3 were considered a cluster. The term that was the most statistically significant in a cluster is chosen as the one to represent the cluster (52).

### Patient populations and sample collection.

The 2022 Global Strategy for Asthma Management and Prevention was employed for asthma diagnosis (53). Asthma was categorized as MMA and SA by disease severity described in a previous study (33). Sputum samples, pulmonary function tests (within 14 days before and after sample collection), and medication records were processed or collected from 21 HCs, 33 patients with MMA, and 6 patients with SA at Xiangya Hospital of Central South University. Among them, 7 patients with asthma lacked pulmonary function test data and 5 patients with asthma lacked ACT score data.

### Quantitative CT assessment of airways.

The computed tomography (CT) scans of patients with asthma were obtained from the Imaging Department of Xiangya Hospital of Central South University. 3D reconstruction of airway trees was conducted and the airway dimensions were assessed as previously described (33). Among them, 16 patients with asthma lacked airway CT parameters data.

### Mice.

All animal study methods were carried out in accordance with the relevant guidelines and regulations. For rmCTSC-challenged (CSB-EP006186MO, Cusabio) mice, WT mice (background, C57BL/6) were administered with 1 μg rmCTSC in 20 μL PBS intranasally, 3 times per week for 3 weeks (54).

Asthma models with different degrees of airway remodeling were constructed according to previous publications with minor modifications (55, 56). For the HDM-induced asthma model, 6- to 8-week-old WT mice (CTSC^+/+^) or CTSC-knockout mice (CTSC^–/–^, provided by Christine Pham of Washington University in St. Louis, St. Louis, Missouri, USA) were given 50 μg HDM (in 20 μL PBS; XPB91D3A25, Greer Laboratories) or PBS intranasally, 3 times a week for 7 weeks. For the SA model, WT mice were sensitized with 25 μg HDM combined with 5 μg c-di-GMP (in 20 μL PBS; 61093-23-0, Invivogen) intranasally on days 1, 3, and 5. Mice were rested for 5 days and then subjected to 3 repeated challenges. Specifically, mice were given 0.5 μg c-di-GMP combined with 25 μg HDM (in 20 μL PBS) intranasally on days 11, 18, and 25 and were given 25 μg HDM (in 20 μL PBS) intranasally on days 12, 13, 19, 20, 26, and 27 (55). For some groups, dexamethasone (4 mg/kg) was given i.p. starting on the first day of the challenge and then repeated every third day. For AZD7986 (HY-101056, MCE) treatment, HDM-induced mice were treated orally with AZD7986 (5 mg/kg) or vehicle controls (corn oil) once per week 1 hour before HDM challenge (57).

### Histopathology and immunohistochemistry of lung tissue.

Lungs were inflated, fixed in 4% paraformaldehyde, and embedded in paraffin blocks that were cut into 5 μm sections. H&E staining was performed and used to evaluate airway inflammation and the thickness of basement membrane (58). Periodic acid-Schiff (PAS) stain (Shanghai Sun Biotechnology) was used to detect muco-substances in the airway epithelium following manufacturer’s instructions. The number of PAS-positive epithelial cells in individual bronchioles was counted blindly as previously described (59). Masson’s trichrome staining was utilized to measure collagen deposition of small airway (60). Immunohistochemistry staining was applied with the following antibodies: CTSC antibody (AF1034-SP, R&D Systems), Ki67 antibody (WL01384a, Wanleibio), E-cadherin (E-cad) antibody (sc-8426, Santa Cruz Biotechnology), vimentin antibody (5741S; Cell Signaling Technology), α-SMA antibody (ab5694, Abcam), and Muc5ac antibody (K010199P, Solarbio). The primary antibody was omitted in negative controls. Quantification of Masson’s trichrome staining and immunohistochemistry staining was performed by using Image Pro-Plus software version 4.5 (Media Cybernetics). The above pathological assessments were all adjudicated by 2 blinded technicians.

### Cell cultures and stimulation.

HBECs from the same sample were purchased from Lifeline Cell Technology. An immortalized HBEC line (16HBE14o- cells) was presented by the University of California at San Francisco. HBECs and 16HBE14o- cells were cultivated in DMEM with 10% FBS and incubated at 37°C in 5% CO_2_. For the induction of HDM stimulation, cells were treated with HDM (75 mg/mL) for 0 hours, 24 hours, 48 hours, and 72 hours.

Human lung fibroblasts (HLF-1) were obtained from ATCC and cultivated in HAM’S F-12K complete medium with 10% FBS. Recombinant human CTSC (rhCTSC, 1071-CY-010, R&D Systems) was activated according to the manufacturer’s instruction (25). HLF-1 cells were stimulated with rhCTSC for 48 hours. In the group with agonist treatment, some HLF-1 were pretreated with SP203580 (p38 phosphorylation inhibitor, 5 mmol/L, S1076, Selleck) for 30 minutes and then stimulated with activated rhCTSC for 48 hours.

### Western blot analysis.

Western blot analysis was performed under the previous procedures (33). In brief, 50 μg protein isolated from HBECs or HLF-1 cells was separated by 10% SDS-PAGE and transferred to a PVDF membrane. Then, the PVDF membrane was incubated with primary antibody for 12 hours and next incubated with HRP-conjugated secondary antibody. CTSC antibody (sc-74590, Santa Cruz Biotechnology), α-SMA antibody (ab5694, Abcam), COL 1 antibody (ab34710, Abcam), p38 antibody (IPB5239, Taizhou Baijia Biotechnology), p-p38 antibody (IPH1206, Taizhou Baijia Biotechnology), Erk antibody (ab184699, Abcam), p-Erk antibody (ab4819, Abcam), JNK antibody (sc-7345, Santa Cruz Biotechnology), p-JNK antibody (sc-6254, Santa Cruz Biotechnology), and β-tubulin antibody (10068-1-AP, Proteintech) were used to assess the level of the corresponding protein with Western blot analysis.

### ELISA.

The level of CTSC in the supernatants of HBECs was determined with ELISA assays according to the manufacturer’s protocols (SEC965Hu, Cloud-Clone Corp).

### Construction and transfection of plasmid.

CTSC overexpression vector, silence vector, and corresponding control vector were acquired from Genechem Co., Ltd. For stable transfection, HBECs were transfected with different vectors using Lipofectamine 3000 and P3000 (Thermo). Transfection efficiency was confirmed by real time RT–PCR and Western blot analysis.

### RNA extraction, RT-PCR and quantitative RT-PCR.

Total mRNA was purified from transfected cells using RNAiso Plus (TaKaRa Clontech). RNA was reversely transcribed into cDNA with the PrimeScript RT Reagent Kit (TaKaRa Bio). Quantitative RT-PCR was performed using the SYBR Premix Ex Taq II system (TaKaRa) with the CFX96 Touch Real-Time PCR Detection System (Bio-Rad). The PCR conditions were as follows: 95°C for 30 seconds, 40 cycles of 95°C for 15 seconds, and 60°C for 30 seconds. By comparing the copy numbers of the target gene and GAPDH, we managed to normalize the mRNA expression data for sample-to-sample variability in RNA input, RNA quality, and the reverse transcription efficiency (61).

### Cell Counting Kit-8 assay.

Cell Counting Kit-8 (CCK-8) assay was performed (Dojindo). HBECs or 16HBE14o- cells in different groups were inoculated in 96-well plates at a density of 5 × 10^3^ cells/mL, and 100 μL culture medium was added into each well. 10 μL CCK-8 reagent was added to each well and then incubated in 5% CO_2_ at 37°C for 2 hours or 4 hours. Finally, the optical density (OD) was measured by a microplate reader (Thermo, Varioskan Flash) at 450/630 nm.

### Growth curves.

Cells were seeded in 96-well plates at a certain number of cells per well counted at 24 hours, 48 hours, 72 hours, and 96 hours using a particle counter (Beckman Coulter).

### Scratch test.

After digesting into a single-cell suspension, transfected HBECs or 16HBE14o- cells were inoculated into 12-well plate at a concentration of 5 × 10^5^ cells/well overnight and scratched vertically with a 200 μL micro pipette tip on the next day. Then, cells were washed twice with PBS and placed in serum-free culture medium. Images were taken at different time points with microscope and analyzed with ImageJ Software (NIH).

### Antioxidation assay.

The antioxidation ability of HBECs or 16HBE14o- cells was evaluated as previously described (62).

### Permeability measurements.

Permeability measurements were carried out as previously described (63, 64) with minor modifications. Briefly, HBECs or 16HBE14o- cells grew until complete confluence, and 200 μL phenol red-free DMEM containing 0.5 mg/mL FITC-labeled dextran (Sigma Chemical Co.; molecular weight 77,000 mol) was added to the apical compartments (luminal side). Then, 600 μL phenol red-free DMEM without FITC-labeled dextran was added to the basal compartments (nonluminal side). Cultures were allowed to equilibrate at 37°C for 90 minutes. FITC fluorescence of basal medium samples was measured by a microplate reader.

### Immunofluorescence staining.

Immunofluorescence staining of HBECs or 16HBE14o- cells was performed using the following antibodies: E-cad antibody (sc-8426, Santa Cruz Biotechnology) and ZO-1 antibody (21773-1-AP, Proteintech) under the previous procedures (65).

### Statistics.

All data were analyzed with GraphPad Prism Software (version 6). Characteristics of patients with asthma were analyzed using χ^2^ test, Fisher’s exact test, or Mann-Whitney *U* test. Pearson correlation was used to assess the associations among the expression of CTSC and lung function or airway CT parameters. The area under the receiver operating characteristic (AUC/ROC) curves was used to evaluate the predictive efficacy of CTSC expression. Comparisons between 2 groups were performed with Mann-Whitney *U* test or unpaired 2-tailed *t* test. Differences in means between multiple groups were examined by 1-way ANOVA followed by Tukey’s post hoc test or 2-way ANOVA followed by Tukey’s post hoc test. *P* values of less than 0.05 were considered significant.

### Study approval.

This study was approved by the Institutional Review Board of the School of Basic Medical Science of Central South University (no. 2020KT-51). All necessary informed consents were obtained from all patients in writing for permission to use their clinical information and samples for analysis. The animal studies were approved by the Central South University at XiangYa Animal Care and Use Committee (no. 2020sydw0305).

### Data availability.

Microarray data are accessible in the GEO database through GEO Series accession GSE19187 (https://www.ncbi.nlm.nih.gov/geo/query/acc.cgi?acc=GSE19187) and GSE63142 (https://www.ncbi.nlm.nih.gov/geo/query/acc.cgi?acc=GSE63142). Values for all data points in graphs are reported in the Supporting Data Values file.

## Author contributions

LY carried out the experiments, analyzed and interpreted the data, and drafted the manuscript. Qingwu Qin, YY, LC, HL, XD, MJ, XW, WW, and Qiuyan Qin performed the experiments and statistical analysis. YX, BQ, X Qu, MY, and X Qin analyzed and interpreted the data, provided the project funding, and revised the manuscript. ZX and CL analyzed and interpreted the data, revised the manuscript, and approved the version of the manuscript for publication. All authors provided critical feedback and helped shape the research, analysis and manuscript.

## Supplementary Material

Supplemental data

Unedited blot and gel images

Supporting data values

## Version 1 10/22/2024

In-Press Preview

## Version 2 11/22/2024

Electronic publication
