# Supplementary material for: Increased expression of cathepsin C in airway epithelia exacerbates airway remodeling in asthma
Source: JCI Insight. 2024 Nov 22;9(22):e181219. doi: 10.1172/jci.insight.181219 (PMC11601913; doi:10.1172/jci.insight.181219)
Supplement: Supplemental data [file jciinsight-9-181219-s170.pdf]

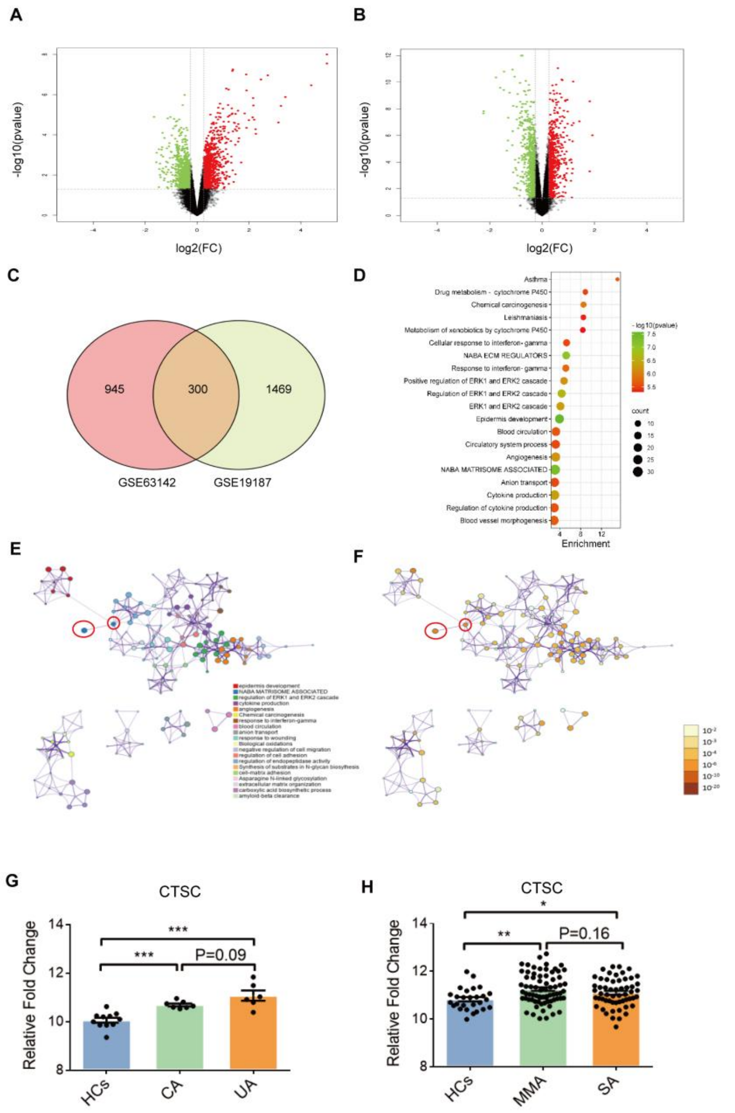

Figure S1. Expression profiles and bioinformatics analysis of DEGs in SA and UA patients. (A) Volcano plot of all genes in GSE19187. The vertical line represented the significance level at P = 0.05. (B) Volcano plot of all genes in GSE63142. (C) Venn diagram comparison of regulated genes between GSE19187 and GSE63142. The genes regulated in common are represented in the region of interaction between yellow and pink circles. (D) Advanced bubble chart shows the enrichment of terms across 300 DEGs. (E) Network of enriched terms colored by cluster ID, nodes that share the same cluster are typically close to each other. (F) Network of enriched terms colored by p-value, terms containing more genes tend to have a more significant *P*-value. The pathways represented by the red circle are NABA MATRISOME ASSOCIATED and NABA ECM REGULATORS respectively. (G) CTSC expression of nasal epithelial samples in HCs (n=11), CA (n=7) and UA (n=6) from GSE19187. (H) CTSC expression of bronchial epithelium samples in HCs (n=27), MMA (n=72) and SA (n=56) from GSE63142. All data presented as mean ± SEM. * $P < 0.05$, ** $P < 0.01$, *** $P < 0.001$ by Unpaired T test.

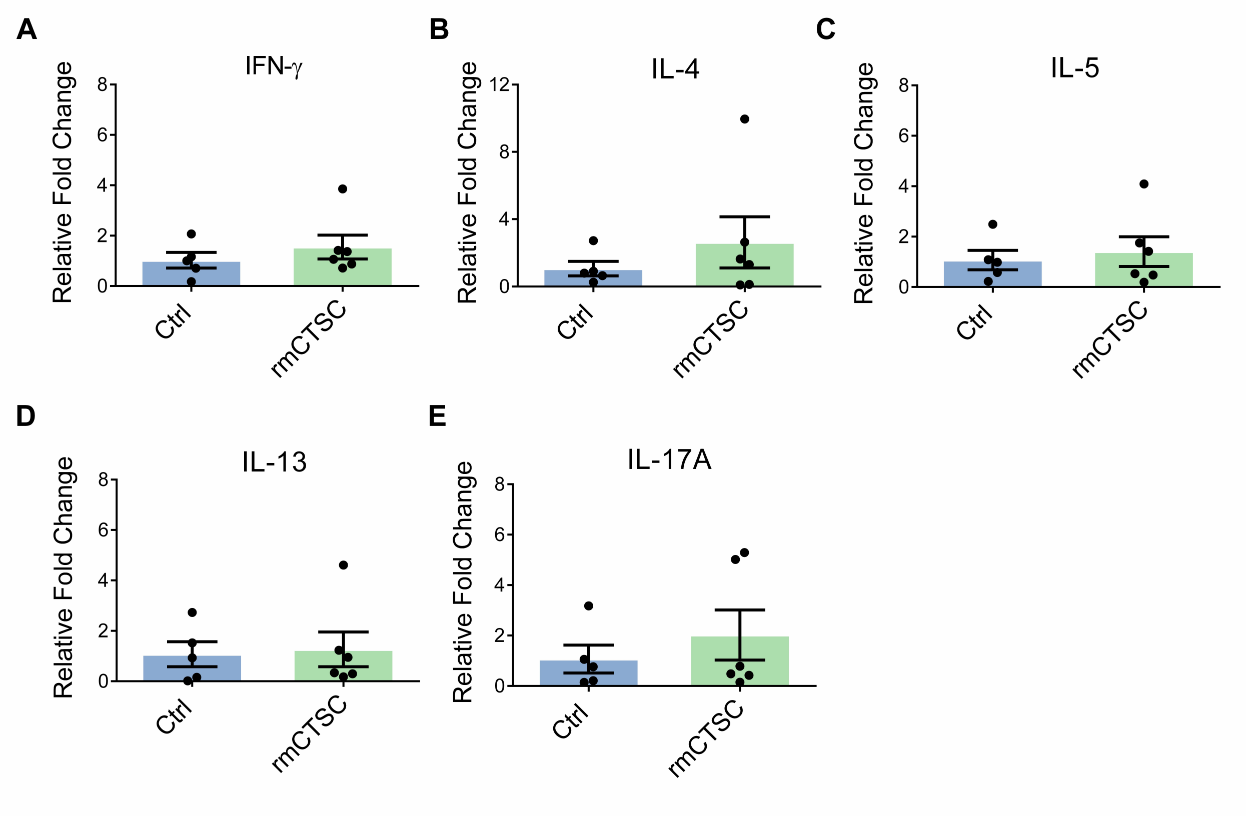

Figure S2. Exogenous CTSC does not affect lung inflammation. (A-E) The levels of IFN-γ, IL-4, IL-5, IL-13 and IL-17A transcripts in lung tissue were examined by quantitative PCR (n=5-6). All data presented as mean ± SEM. Unpaired T test or Mann-Whitney U test was used.

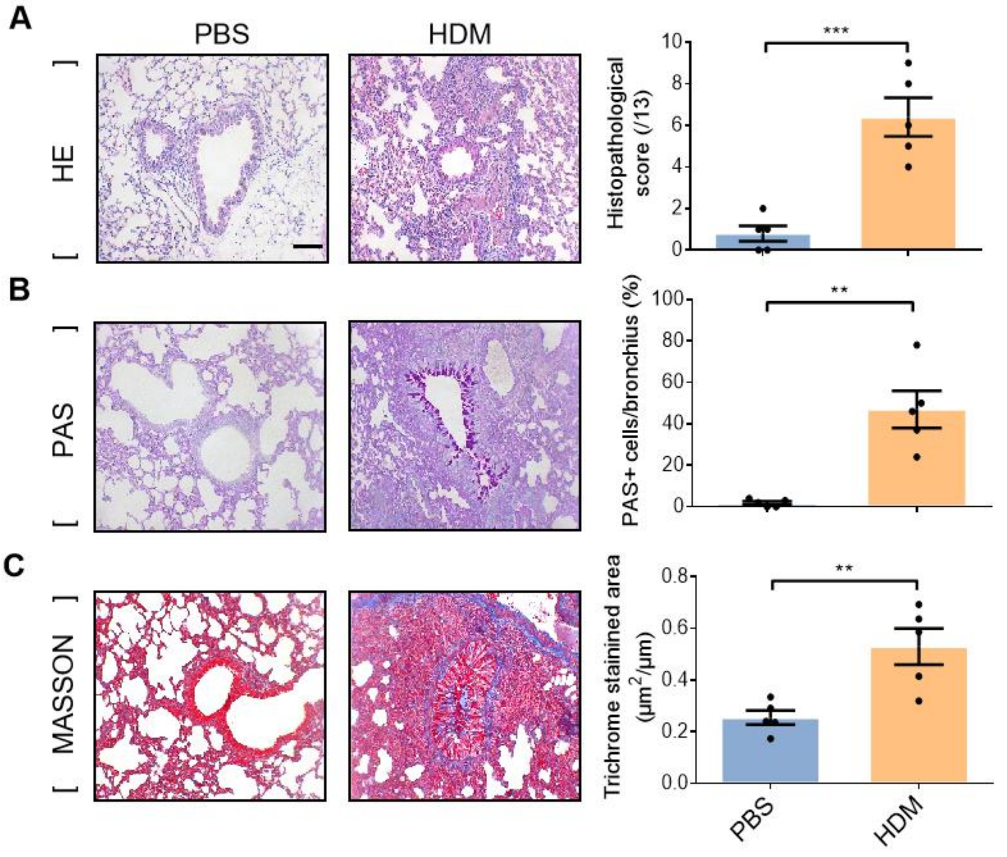

Figure S3. A HDM-induced asthma model with airway remodeling is constructed. (A) Representative lung sections and semiquantitative analysis of airway inflammation (n=5; Scale bar: 50 μm). (B) Representative lung sections and semiquantitative analysis of mucus production (n=5; Scale bar: 50 μm). (C) Representative lung sections and semiquantitative analysis of peribronchial fibrosis (n=5; Scale bar: 50 μm). All data presented as mean ± SEM. ** $P < 0.01$; *** $P < 0.001$ by Unpaired T test or Mann-Whitney U test.

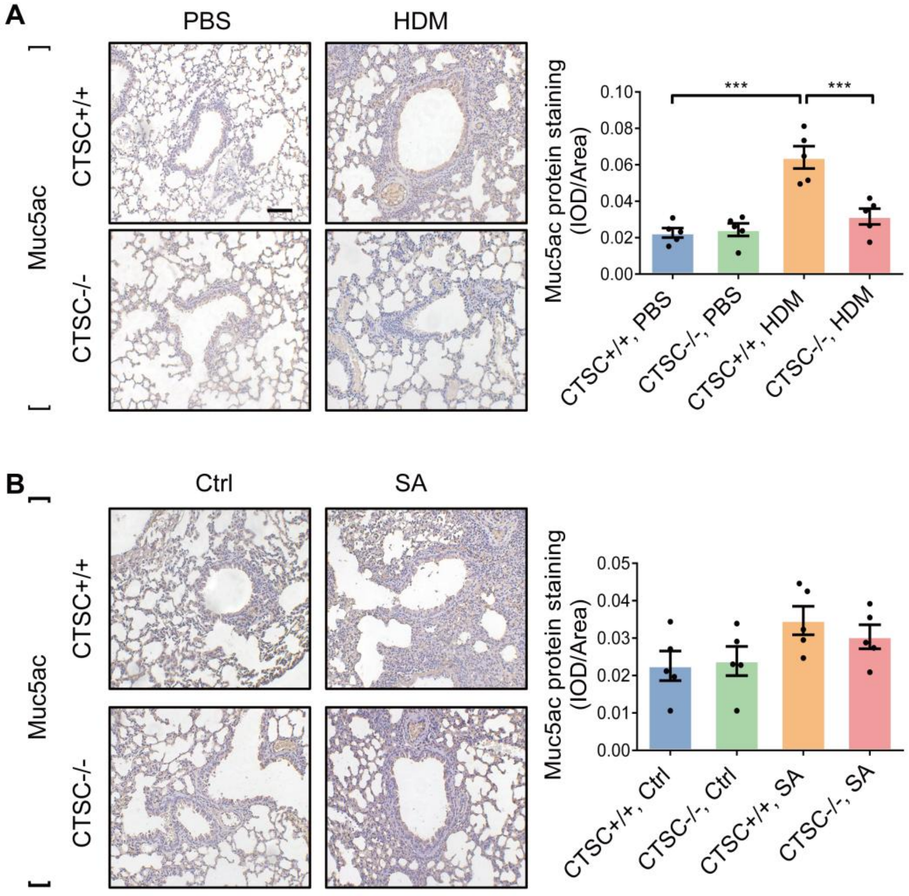

Figure S4. The expression of Muc5ac was examined in HDM-induced asthma model and SA model. (A) Representative immunohistochemistry images of lung tissue and semiquantitative analysis for Muc5ac expression in HDM-induced asthma model (n=5; Scale bar: 50 μm). (B) Representative immunohistochemistry images of lung tissue and semiquantitative analysis for Muc5ac expression in the SA model (n=5; Scale bar: 50 μm). All data presented as mean ± SEM. *** $P < 0.001$ by one-way ANOVA followed by Tukey's post hoc test.

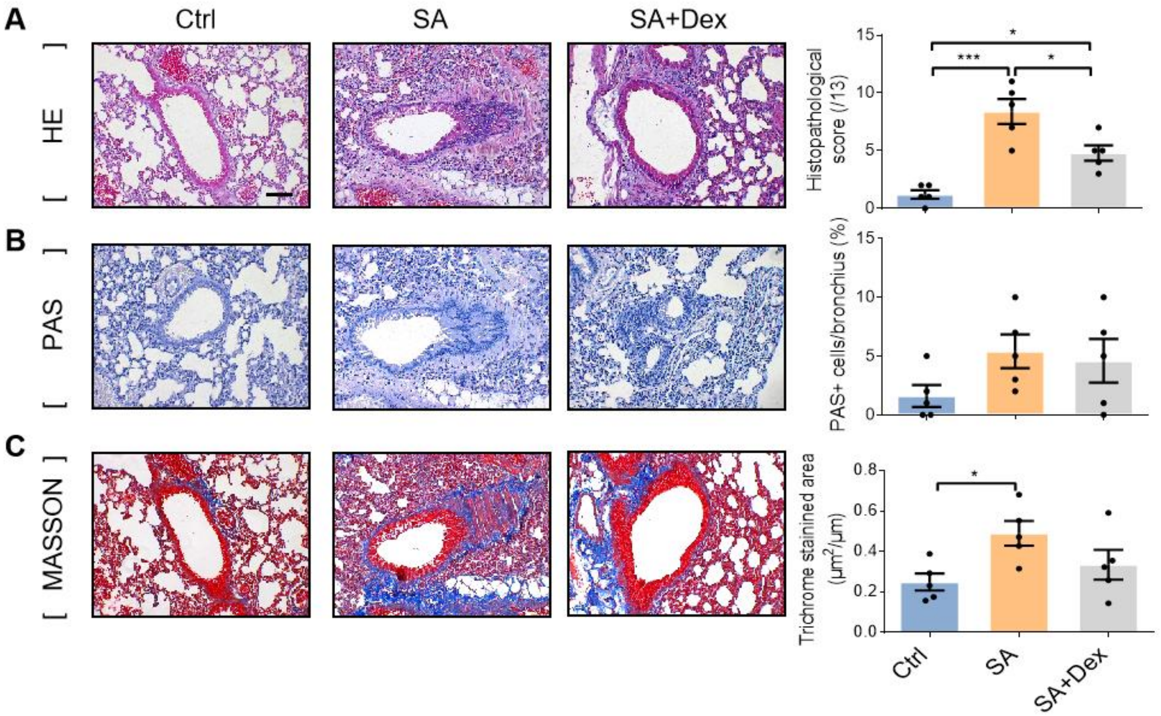

Figure S5. A SA model with airway remodeling is constructed. (A) Representative lung sections and semiquantitative analysis of airway inflammation (n=5; Scale bar: 50 μm). (B) Representative lung sections and semiquantitative analysis of mucus production (n=5; Scale bar: 50 μm). (C) Representative lung sections and semiquantitative analysis of peribronchial fibrosis (n=5; Scale bar: 50 μm). All data presented as mean ± SEM. * $P < 0.05$; ** $P < 0.01$; *** $P < 0.001$ by one-way ANOVA followed by Tukey's post hoc test.

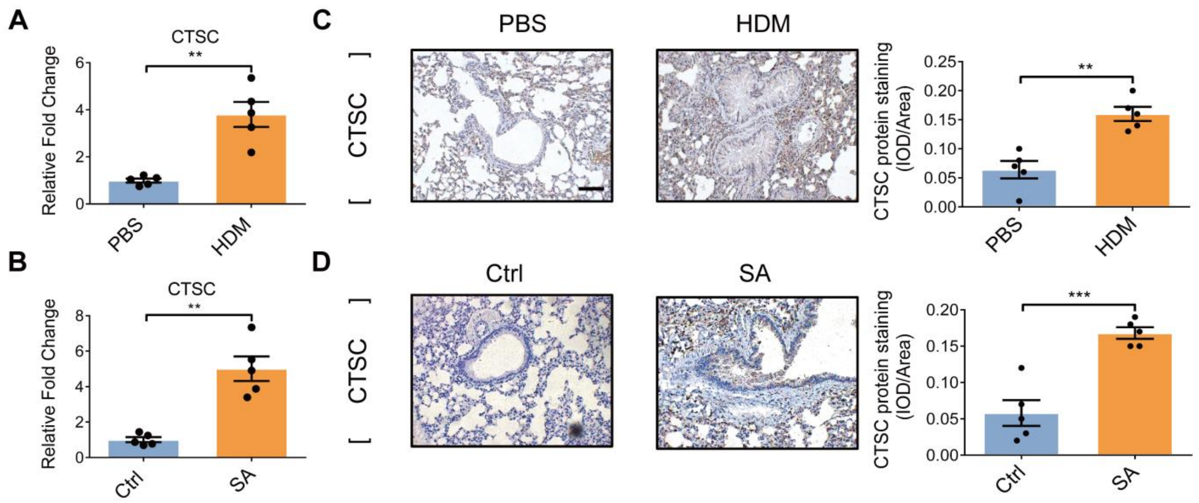

Figure S6. The expression of CTSC in AECs increases markedly after stress. (A, B) The levels of CTSC transcripts in lung tissue of HDM model or SA model were examined by quantitative PCR. (C, D) Immunohistochemistry of CTSC in lung tissue of HDM model or SA model (n=5; Scale bar: 50 μm). All data presented as mean ± SEM. ***P* < 0.01; ****P* < 0.001 by Unpaired T test or Mann-Whitney U test.

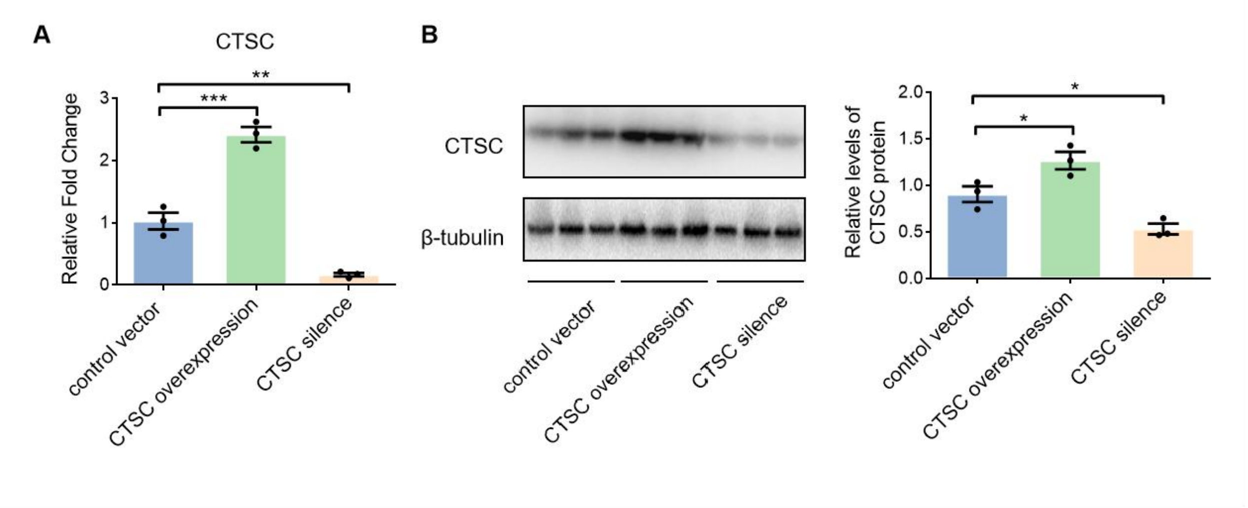

Figure S7. Silencing and overexpression efficiency of CTSC plasmid in HBECs. (A) The expression of CTSC mRNA in HBECs was detected by qPCR 48 hours after transfection (n=3). (B) CTSC protein expression was tested by western blot analysis 48 hours after transfection (n=3). All data presented as mean ± SEM. **P* < 0.05; ***P* < 0.01 by one-way ANOVA followed by Tukey's post hoc test.

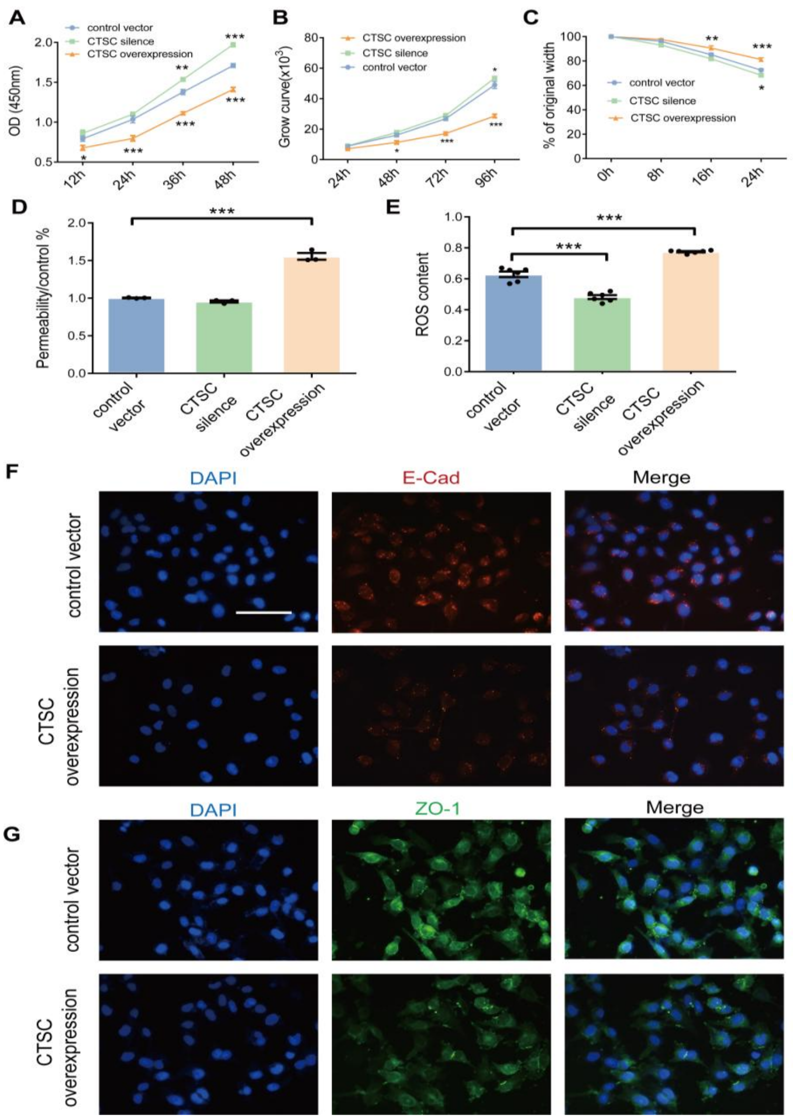

Figure S8. The increased expression of CTSC in 16HBE14o- cells impairs the epithelial barrier function. (A) Cell proliferation analysis of 16HBE14o- cells after CSTC silencing and overexpression using CCK-8 assay (n=6). Two-way ANOVA followed by Tukey's post hoc test was used. (B) Proliferation curves of 16HBE14o- cells after CSTC silencing and overexpression (n=4). Two-way ANOVA followed by Tukey's post hoc test was used. (C) A scratch test for evaluating damage repair capability in 16HBE14o- cells (n=3). Two-way ANOVA followed by Tukey's post hoc test was used. (D) Effects of CTSC expression on the permeability of 16HBE14o- cells monolayers (n=3). One-way ANOVA followed by Tukey's post hoc test was used. (E) Effects of CTSC expression on ROS generation (n=6). One-way ANOVA followed by Tukey's post hoc test was used. (F, G) Representative immunofluorescence of 16HBE14o- cells after CSTC overexpression stained for E-cad and ZO-1 (n=3; Scale bar: 50 μm). All data presented as mean ± SEM. * $P < 0.05$, ** $P < 0.01$, *** $P < 0.001$.

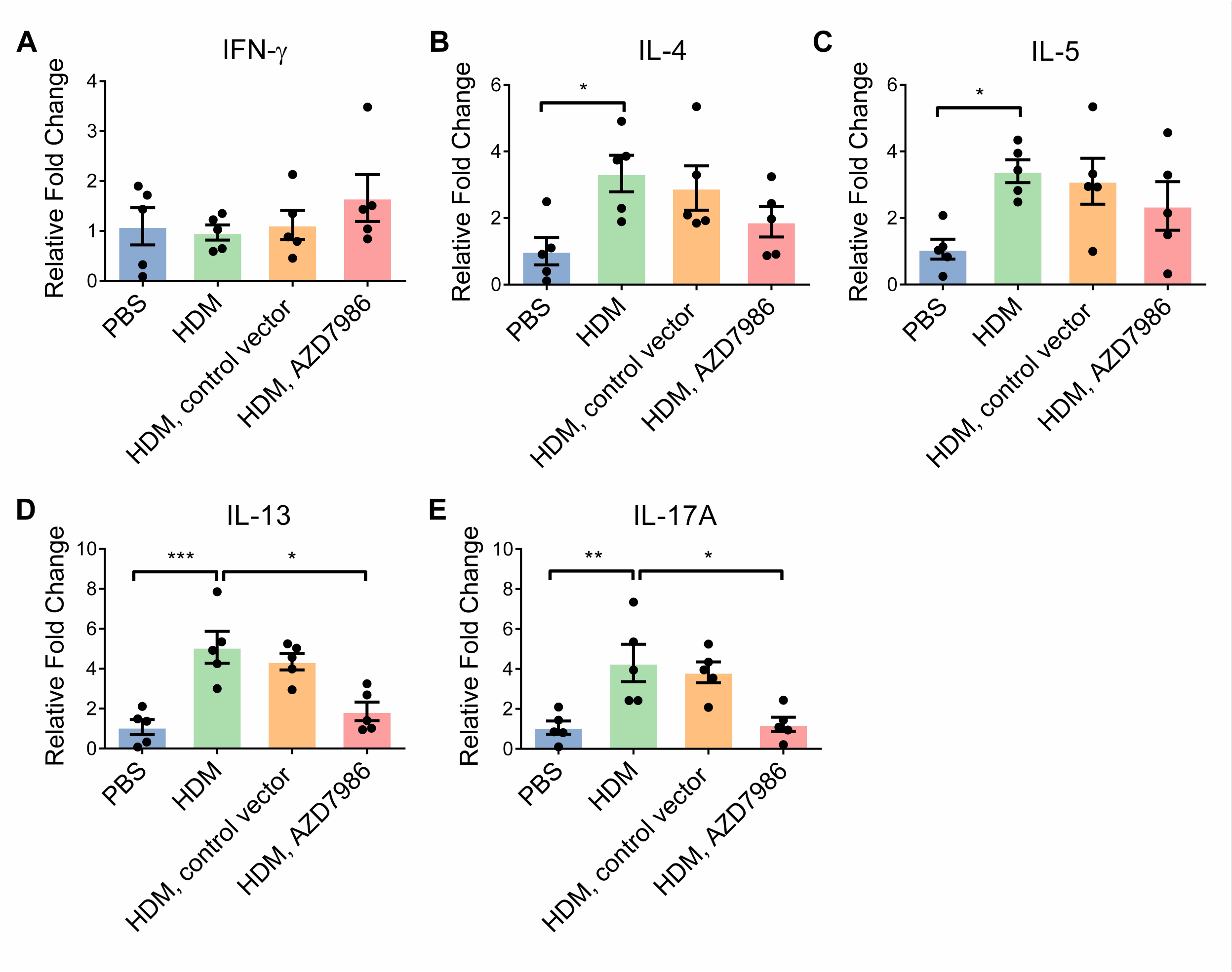

Figure S9. The administration of AZD7986 relieves lung inflammation in HDM-induced asthma model with airway remodeling. (A-E) The levels of IFN-γ, IL-4, IL-5, IL-13 and IL-17A transcripts in lung tissue were examined by quantitative PCR (n=5). All data presented as mean ± SEM. * $P < 0.05$; ** $P < 0.01$; *** $P < 0.001$ by one-way ANOVA followed by Tukey's post hoc test.

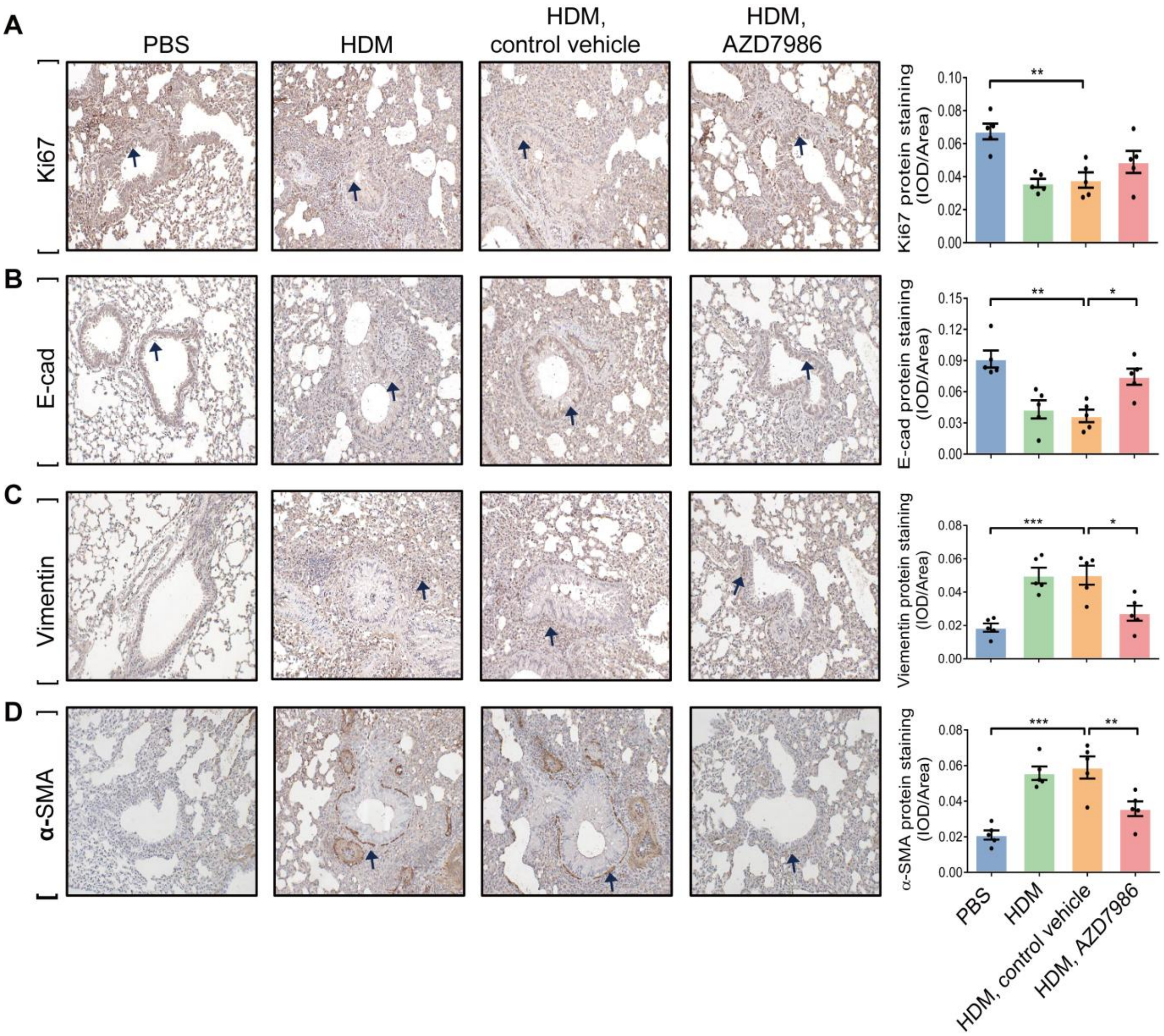

Figure S10. The administration of AZD7986 alleviates the activation of EMTU in HDM-induced asthma model with airway remodeling. (A-D) Representative immunohistochemistry images of lung tissue and semiquantitative analysis for Ki67, E-cad, Vimentin and α-SMA expression (n=5; Scale bar: 50 μm). All data presented as mean ± SEM. * $P < 0.05$; ** $P < 0.01$; *** $P < 0.001$ by one-way ANOVA followed by Tukey's post hoc test.

Table S1. Pathway and process enrichment analysis of differentially expressed genes associated with severe asthma and uncontrolled asthma

Category	GO	Description	LogP	Gene Symbols	Rank
GO Biological Processes	GO:0008544	epidermis development	-7.58	ALOX15B,CST6,CSTA, CTGF,CTSV,FGFR1,GJB5, IGFBP5,INSR,KRT6A	1
Canonical Pathways	M5885	NABA MATRISOME ASSOCIATED	-7.32	CTSC,CLC,CST1,CST2, CST6,CSTA,CTSG, CTSV,SERPINF1,SERPINB8	2
Canonical Pathways	M3468	NABA ECM REGULATORS	-7.06	CTSC,CST1,CST2,CST6, CSTA,CTSG,CTSV,SERPINF1, SERPINB8,SERPINB13	3
GO Biological Processes	GO:0070372	regulation of ERK1 and ERK2 cascade	-6.68	ALOX15,CD36,CD44,CHI3L1, CTGF,DUSP4,EPHB3,FGFR1, INSR,LYN	4
GO Biological Processes	GO:0001816	cytokine production	-6.37	ADRA2A,ALOX15B,C3,C3AR1, CD36,CHI3L1,CLC,CLU,CD55, FGFR1DPB1	5
GO Biological Processes	GO:0070371	ERK1 and ERK2 cascade	-6.33	ALOX15,CD36,CD44,CHI3L1, CTGF,DUSP4,EPHB3,FGFR1, INSR,LYN	6
GO Biological Processes	GO:0001525	angiogenesis	-6.23	C3,C3AR1,CHI3L1,CTGF,EPAS1, EPHB3,FGFR1,HK2,SERPINF1, PTPRMS9,ACKR3,PLXDC1	7
GO Biological Processes	GO:0070374	positive regulation of ERK1 and ERK2 cascade	-6.20	ALOX15,CD36,CD44,CHI3L1, CTGF,EPHB3,FGFR1,INSR, NTRK2,P2RY1	8
KEGG Pathway	hsa05204	Chemical carcinogenesis	-5.96	KYAT1,CYP2C18,EPHX1,GSTA1, GSTA2,GSTA3,GSTM3,HPGDS, GSTA5	9
GO Biological Processes	GO:0034341	response to interferon-gamma	-5.78	CD44,DAPK1,GAPDH,GSN, HLA-DPB1,HLA-DQA1,HLA-DQA2,HLA-DRA,NOS2,CCL20	10
GO Biological Processes	GO:0048514	blood vessel morphogenesis	-5.66	C3,C3AR1,CHI3L1,CTGF,EPAS1, EPHB3,FGFR1,HK2,NTRK2, SERPINF1	11
GO Biological Processes	GO:0008015	blood circulation	-5.63	ADRA2A,C3AR1,CPA3,CTGF, CTSG,EPAS1,F5,HSD11B2, NOS2,P2RY1	12
KEGG Pathway	hsa05310	Asthma	-5.62	MS4A2,HLA-DMB,HLA-DPB1, HLA-DQA1,HLA-DQA2,HLA-DRA	13
GO Biological Processes	GO:0001817	regulation of cytokine production	-5.59	ADRA2A,ALOX15B,C3,C3AR1, CD36,CLC,CLU,FGFR1,GAPDH, HLA-DPB1	14
GO Biological Processes	GO:0006820	anion transport	-5.54	CA12,CCK,CD36,CLCA1,AKR1C1, SLC26A2,FGFR1,NOS2,NTRK2, SLC1A5	15
GO Biological Processes	GO:0003013	circulatory system process	-5.52	ADRA2A,C3AR1,CPA3,CTGF, CTSG,EPAS1,F5,HSD11B2, NOS2,P2RY1	16
GO Biological Processes	GO:0071346	cellular response to interferon-gamma	-5.50	CD44,DAPK1,GAPDH,GSN, HLA-DPB1,HLA-DQA1,HLA-DQA2,HLA-DRA,NOS2,CCL20	17
KEGG Pathway	hsa00982	Drug metabolism - cytochrome P450	-5.49	GSTA1,GSTA2,GSTA3,GSTM3,	18

				MAOA,MAOB,HPGDS,GSTA5	
KEGG Pathway	hsa05140	Leishmaniasis	-5.35	C3,HLA-DMB,HLA-DPB1,<br>HLA-DQA1,HLA-DQA2,<br>HLA-DRA,NOS2,TGFB2	19
KEGG Pathway	hsa00980	Metabolism of xenobiotics by cytochrome P450	-5.31	AKR1C1,EPHX1,GSTA1,GSTA2,<br>GSTA3,GSTM3,HPGDS,GSTA5	20

Table S2. Characteristics of HCs and patients with asthma

	HCs	Asthma	MMA	SA
n	21	39	33	6
Age	40.29±13.26	47.42±12.50	47.38±12.39	47.75±15.35
BMI	22.39±2.89	24.53±3.32	24.60±3.37	23.95±3.20
FEV_1%	/	0.73±0.21	0.77±0.19	0.45±0.09**
FEV_1/FVC	/	0.68±0.18	0.71±0.17	0.48±0.03*
ACT Score	/	16.79±3.78	17.50±3.34	11.50±2.65**
Wall thickness (mm)	/	0.99±0.10	0.96±0.08	1.16±0.03**
Wall thickness (%)	/	42.60±3.31	42.03±3.06	46.40±2.63*
Wall area (mm^2)	/	12.05±1.53	11.63±1.13	14.83±0.32**
Wall area (%)	/	66.91±3.82	66.29±3.59	70.82±3.31

Data are presented as mean ± SD or n.*$P < 0.05$, **$P < 0.01$. Patients with MMA versus those with SA.
