# Supplementary material for: Increased expression of cathepsin C in airway epithelia exacerbates airway remodeling in asthma
Source: JCI Insight. 2024 Nov 22;9(22):e181219. doi: 10.1172/jci.insight.181219 (PMC11601913; doi:10.1172/jci.insight.181219)

Full unedited gel for Figure 6C:

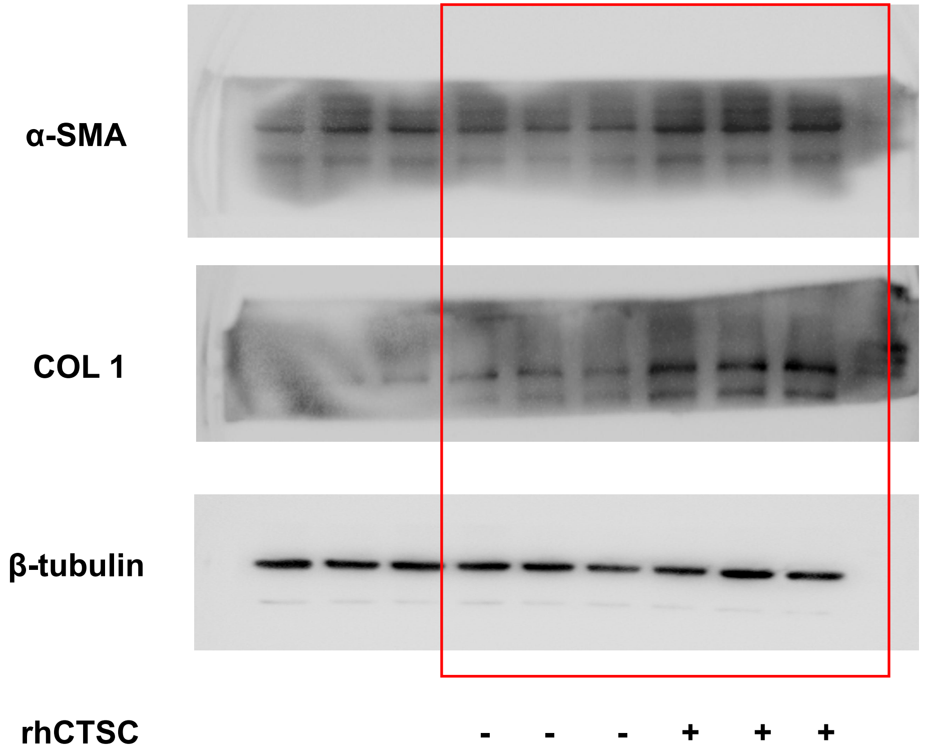

Full unedited gel for Figure 6D：

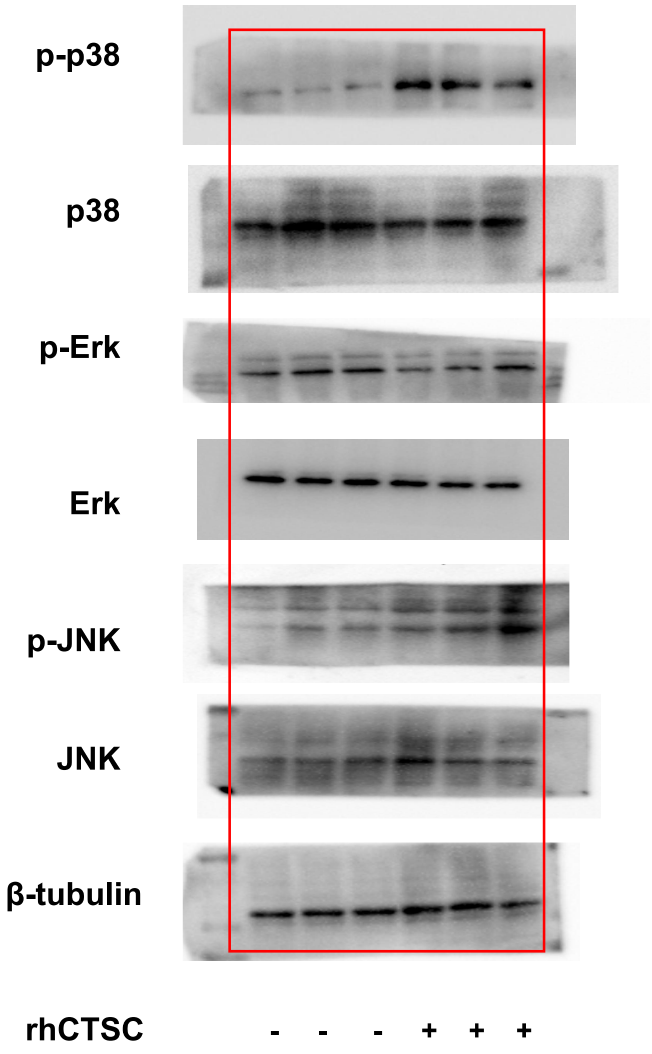

Full unedited gel for Figure 6E：

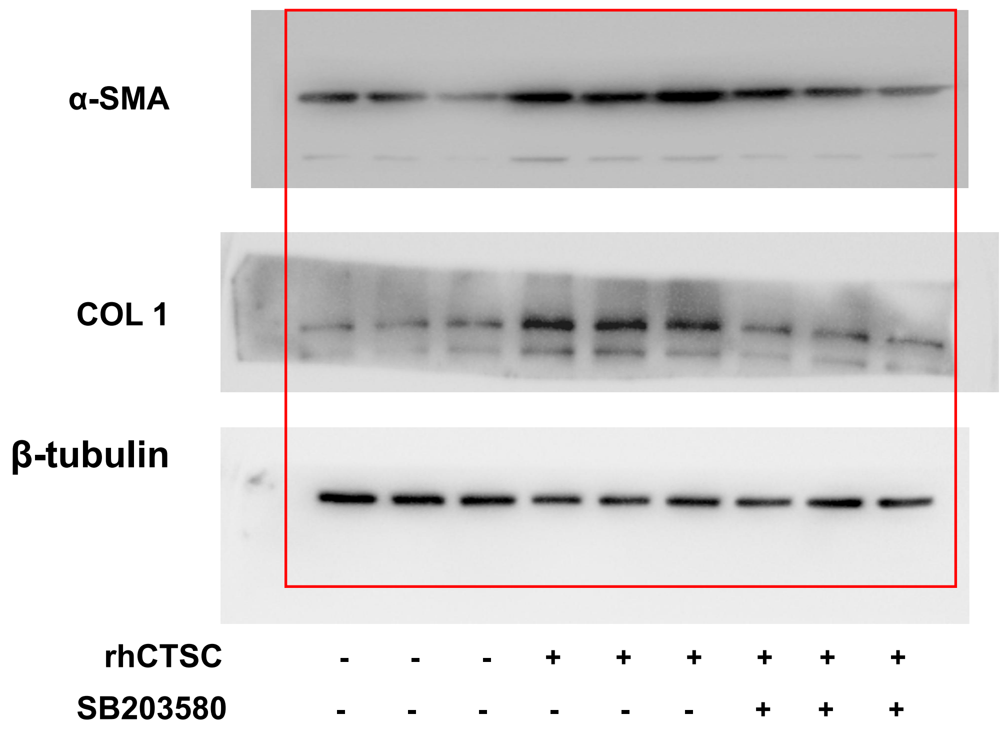

Full unedited gel for Figure S7B

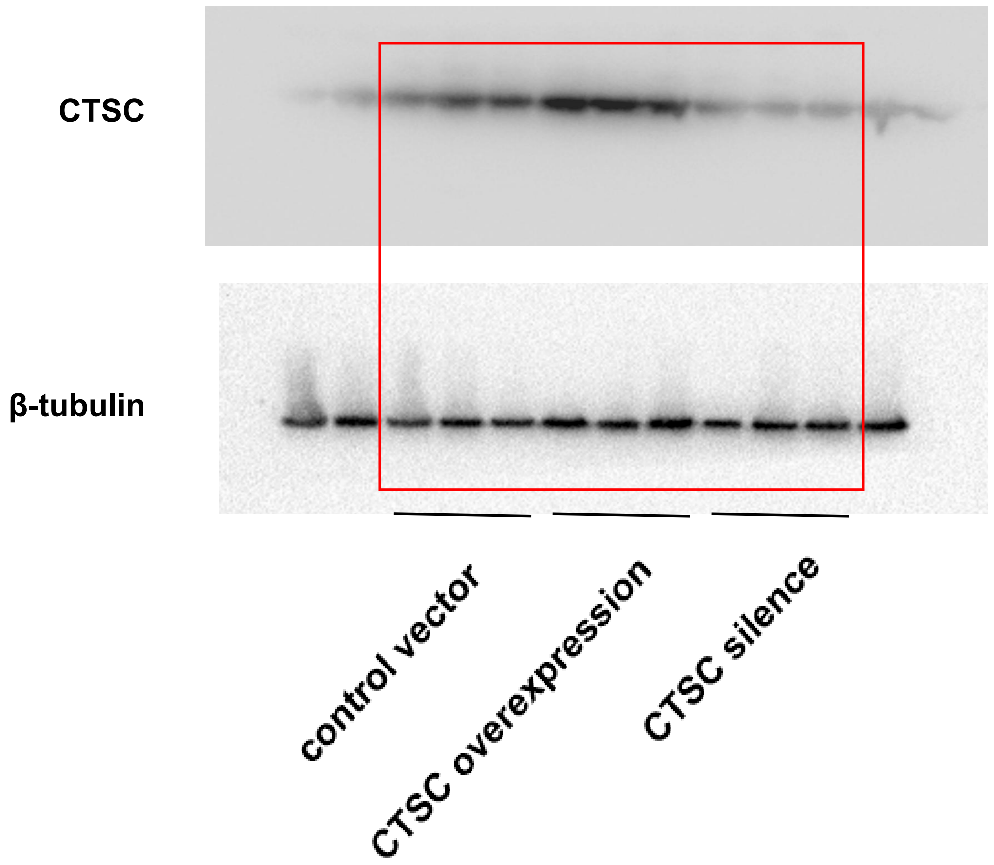

Supplement: Unedited blot and gel images [file jciinsight-9-181219-s171.pdf]
